# ISG15 protects human Tregs from interferon alpha‐induced contraction in a cell‐intrinsic fashion

**DOI:** 10.1002/cti2.1221

**Published:** 2020-12-23

**Authors:** Ilenia Pacella, Francesca Romana Spinelli, Martina Severa, Eleonora Timperi, Gloria Tucci, Marta Zagaglioni, Fulvia Ceccarelli, Fabiana Rizzo, Eliana M Coccia, Roosheel S Patel, Marta Martin‐Fernandez, Dusan Bogunovic, Fabrizio Conti, Vincenzo Barnaba, Silvia Piconese

**Affiliations:** ^1^ Dipartimento di Scienze Cliniche Internistiche, Anestesiologiche e Cardiovascolari Sapienza Università di Roma Rome Italy; ^2^ Department of Infectious Diseases Istituto Superiore di Sanità Rome Italy; ^3^ Laboratory affiliated to Istituto Pasteur Italia‐Fondazione Cenci Bolognetti Rome Italy; ^4^ Center for Inborn Errors of Immunity Icahn School of Medicine at Mount Sinai New York NY USA; ^5^ Precision Immunology Institute Icahn School of Medicine at Mount Sinai New York NY USA; ^6^ Mindich Child Health and Development Institute Icahn School of Medicine at Mount Sinai New York NY USA; ^7^ Department of Pediatrics Icahn School of Medicine at Mount Sinai New York NY USA; ^8^ Department of Microbiology Icahn School of Medicine at Mount Sinai New York NY USA; ^9^ Present address: Eleonora Timperi Institut Curie Paris France

**Keywords:** hepatitis C, interferon, ISG15, lupus, STAT1, Tregs

## Abstract

**Objectives:**

Type I interferons (IFNs) inhibit regulatory T‐cell (Treg) expansion and activation, making them beneficial in antiviral responses, but detrimental in autoimmune diseases. Herein, we investigate the role of ISG15 in human Tregs in the context of refractoriness to type I IFN stimulation.

**Methods:**

ISG15 expression and Treg dynamics were analysed *in vitro* and *ex vivo* from patients with chronic hepatitis C, with lupus and ISG15 deficiency.

**Results:**

ISG15 is expressed at high levels in human Tregs, renders them refractory to the IFN‐STAT1 signal, and protects them from IFN‐driven contraction. *In vitro*, Tregs from healthy controls upregulate ISG15 upon activation to higher levels than conventional CD4 T cells, and ISG15‐silenced Tregs are more susceptible to IFNα‐induced contraction. In human ISG15 deficiency, patient Tregs display an elevated IFN signature relative to Tregs from healthy control. *In vivo*, in patients with chronic hepatitis C, 2 days after starting pegIFN/ribavirin therapy, a stronger ISG15 inducibility correlates with a milder Treg depletion. *Ex vivo*, in systemic lupus erythematosus patients, higher levels of ISG15 are associated to reduced STAT1 phosphorylation in response to IFNα, and also to increased frequencies of Tregs, characterising active disease.

**Conclusion:**

Our results reveal a Treg‐intrinsic role of ISG15 in dictating their refractoriness to the IFN signal, thus preserving the Treg population under inflammatory conditions.

## Graphical abstract

Tregs are particularly sensitive to the detrimental effects of type I IFNs. Here, we show that an IFN‐stimulated gene, ISG15, mediates Treg refractoriness to IFN‐induced contraction *in vitro*. In lupus patients, high ISG15 expression sustains high Treg frequencies in active disease. In human ISG15 deficiency, Tregs show an elevated IFN signature.

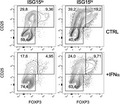

## Introduction

Regulatory T cells (Tregs) are devoted to the suppression of potentially harmful immune responses and to the recovery of immune and tissue homeostasis after injuries.^1^ To accomplish this function, Tregs rapidly adjust their activities in response to diverse inflammatory signals. These cells constitutively express a variety of receptors for soluble mediators including cytokines. During immune responses, different cytokines may have opposite effects on Treg expansion, function or stability, thus finely tuning their suppression depending on the microenvironmental demands.^2^

Following pathogen recognition, almost all the cells in the body are able to synthesise and release type I IFN, which in a paracrine manner promotes antiviral resistance through the induction of an array of IFN‐stimulated genes (ISGs). However, dysregulation of the type I IFN signalling may play detrimental roles in host defence, promoting immunopathology or even inducing immune suppression.^3^ The pathogenic roles of type I IFNs in several autoimmune diseases, and especially systemic lupus erythematosus (SLE), are well established, as documented by the appearance of an IFN‐related gene signature in peripheral blood (PB) concomitantly with disease reactivation.^4^

ISG15 is a ubiquitin‐like protein and one of the most strongly and rapidly induced ISGs.^5^ In its unconjugated form, extracellular ISG15 can act like a cytokine, inducing IFNγ production by lymphocytes. Additionally, ISG15 can be covalently linked to cellular proteins through a process called ISGylation, although exact role for this process in humans is still debated.^5^, ^6^ However, ISG15 displays several immunomodulatory functions: for instance, in chronic hepatitis C (CHC), a persistent expression of ISG15 is responsible for resistance to IFN‐based therapy and contributes to limit inflammation.^7^, ^8^, ^9^, ^10^ Notably, humans with inherited ISG15 deficiency display pathological signs of hypersensitivity to type I IFNs: indeed, in humans, free intracellular ISG15 binds and stabilises Ubiquitin Specific Peptidase 18 (USP18), a negative regulator of type I IFN signalling pathway.^11^ An elevated IFN signature and increased STAT1 phosphorylation in monocytes were probably responsible for driving inflammation in ISG15‐deficient subjects.^12^

It could be hypothesised that hypersensitivity to type I IFNs may compromise the physiological response of Tregs to inflammation due to a stronger Treg‐intrinsic IFN‐STAT1 signal. Indeed, experiments in mouse models of viral infection and cancer have demonstrated that type I IFNs directly restrain Treg proliferation and function, thus allowing the arousal of protective immunity.^13^, ^14^ We have previously shown that, in CHC patients undergoing antiviral therapy with pegIFN/ribavirin (the standard treatment for CHC before the advent of direct‐acting antivirals^15^), Tregs decline *in vivo* as early as after 2 days from therapy starting, in association with increased STAT1 phosphorylation.^16^

We found that such contraction was weaker in those patients experiencing stronger ISG15 induction in peripheral blood mononuclear cells (PBMCs): this observation led us to recognise ISG15 as a molecule protecting Tregs, in a cell‐intrinsic fashion, from excessive IFN‐induced decrease. Confirming this idea, we found the ISG15 protein was highly expressed in activated Tregs and further increased upon IFNα exposure. Importantly, ISG15‐silenced Tregs were more susceptible to IFNα‐induced contraction *in vitro*, and patients with ISG15 deficiency display stronger IFN signature in Tregs as compared to WT controls and expected steady‐state Treg numbers. In SLE patients, high ISG15 expression was associated with high Treg frequencies upon *ex vivo* stimulation, further supporting the notion that ISG15 may dictate Treg refractoriness to the detrimental effects of type I IFNs during inflammation.

## Results

### ISG15 induction correlates with reduced Treg depletion in CHC patients undergoing IFN therapy

**Figure 1:**
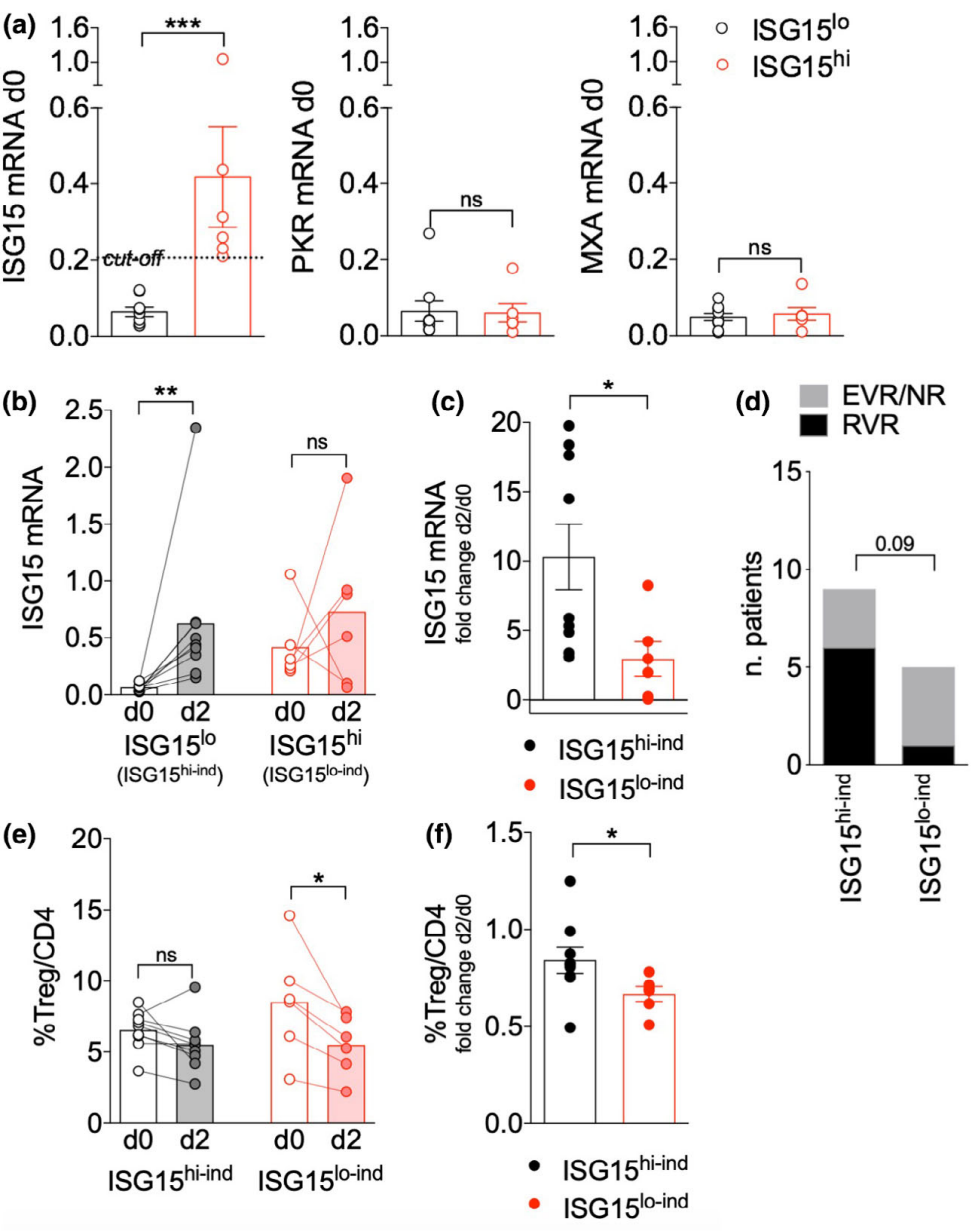
ISG15 induction correlates with protection from Treg depletion in response to IFN therapy in chronic hepatitis C (CHC) patients. **(a**) Real‐time PCR for *ISG15*, *PKR* and *MXA* was performed in peripheral blood mononuclear cells (PBMCs) from 15 CHC patients at day 0 (before starting pegIFN/ribavirin therapy), and patients were stratified into ISG15^lo^ (*n* = 9) and ISG15^hi^ (*n* = 6) using the average ISG15 expression level (0.206) as cut‐off. Means ± SEM of *ISG15*, *PKR* and *MXA* expression levels (2^−ΔCt^ relative to *GAPDH*) are shown in the two subgroups. ***P < 0.005, by the Mann–Whitney *U‐*test; ns, not significant. **(b)** ISG15 expression was assessed in PBMCs of ISG15^lo^ (ISG15^hi‐ind^) and ISG15^hi^ (ISG15^lo‐ind^) CHC patients, stratified as detailed above, at day 0 (day 0) and day (day 2) of pegIFN/ribavirin therapy. **P < 0.01, by the Wilcoxon matched‐pairs test; ns, not significant. **(c)** Fold change in *ISG15* mRNA was calculated as the day 2/day 0 ratio. *P < 0.05, by the Mann–Whitney *U‐*test. **(d)** The chi‐square test was applied to analyse the difference between ISG15^hi‐ind^ and ISG15^lo‐ind^ in terms of rates of RVR versus EVR/NR, stratified according to the response to pegIFN/ribavirin therapy at short‐term follow‐up as previously described.^16^ **(e)** Treg frequency was evaluated in CHC patients, at day 0 and day 2 of therapy, as the percentage of FOXP3^+^ CD127^lo^ in gated live CD14^−^ CD4^+^ PBMCs. *P < 0.05, by the Wilcoxon matched‐pairs test; ns, not significant. **(f)** Fold change in Treg frequency was calculated as the day 2/day 0 ratio. *P < 0.05, by the Mann–Whitney *U‐*test.

We have previously observed that, in CHC patients, Tregs declined as early as 2 days after pegIFN/ribavirin therapy starting and that the induction of several ISGs could be already detected at the same time point in PBMCs.^16^ Among the tested ISGs, we noticed that *ISG15* expression was more variable among patients at baseline (day 0) compared to Protein Kinase R (*PKR*) and Myxovirus resistance protein 1 (*MXA*) (Figure 1a), in line with previous data.^9^ Indeed, the standard deviation (SD) of *ISG15* was 0.265, higher than the SD of *MXA* (0.033), and of *PKR* (0.071). Therefore, we stratified patients into ISG15^lo^ or ISG15^hi^ depending on the expression level of *ISG15* mRNA in total PBMCs with respect to the average value as cut‐off. The two subgroups did not significantly differ in terms of HCV viraemia, ALT/AST levels, or in the subsequent response to pegIFN/ribavirin (not shown). Notably, *PKR* and *MXA* expression remained very low in both groups (Figure 1a). This result suggests that *ISG15* expression may be differently regulated compared to other ISGs and that some CHC patients may display a basally sustained, and likely chronic, ISG15 expression in their circulating cells.

A basal level of ISG15 has been associated to refractoriness to IFN stimulation and control of inflammation.^6^, ^9^, ^17^ In line with these data, the induction of ISG15 2 days after therapy starting (day 2), compared to day 0, was significantly higher in PBMCs of ISG15^lo^ compared to ISG15^hi^ patients (Figure 1b and c). The two patient subgroups were thus defined as ISG15 high‐induction (ISG15^hi‐ind^) and ISG15 low‐induction (ISG15^lo‐ind^), respectively. Indeed, despite the stratification of the two patient subgroups according to ISG15 mRNA at baseline, ISG15mRNA was similar at day 2, denoting a statistically significant difference in its induction. Of note, the fold changes of the other two ISGs, *PKR* and *MXA*, were not significantly different between ISG15^hi‐ind^ and ISG15^lo‐ind^ patients (not shown). The ISG15‐related refractoriness may impact on the clinical response to therapy: indeed, the rate of RVRs (rapid virological responders) with respect to EVRs (early virological responders) or NRs (non‐responders)^16^ tended to be higher in the ISG15^hi‐ind^ compared to the ISG15^lo‐ind^ subgroup (Figure 1d).

We then analysed the changes of Treg frequency in these two patient subgroups: ISG15^hi‐ind^ patients experienced a significantly lower Treg decrease from day 0 to day 2 of pegIFN/ribavirin therapy (Figure 1e and f). These results indicate that a higher basal level of ISG15 expression in PBMCs may be associated to lower induction of this gene following an acute *in vivo* exposure to IFN. More importantly, a weaker ISG15 increase (in total PBMCs) corresponded to a stronger reduction of Treg frequency from day 0 to day 2, suggesting that ISG15 induction may antagonise IFN‐driven Treg decrease.

### Resting Tregs express ISG15 and upregulate it upon activation

**Figure 2:**
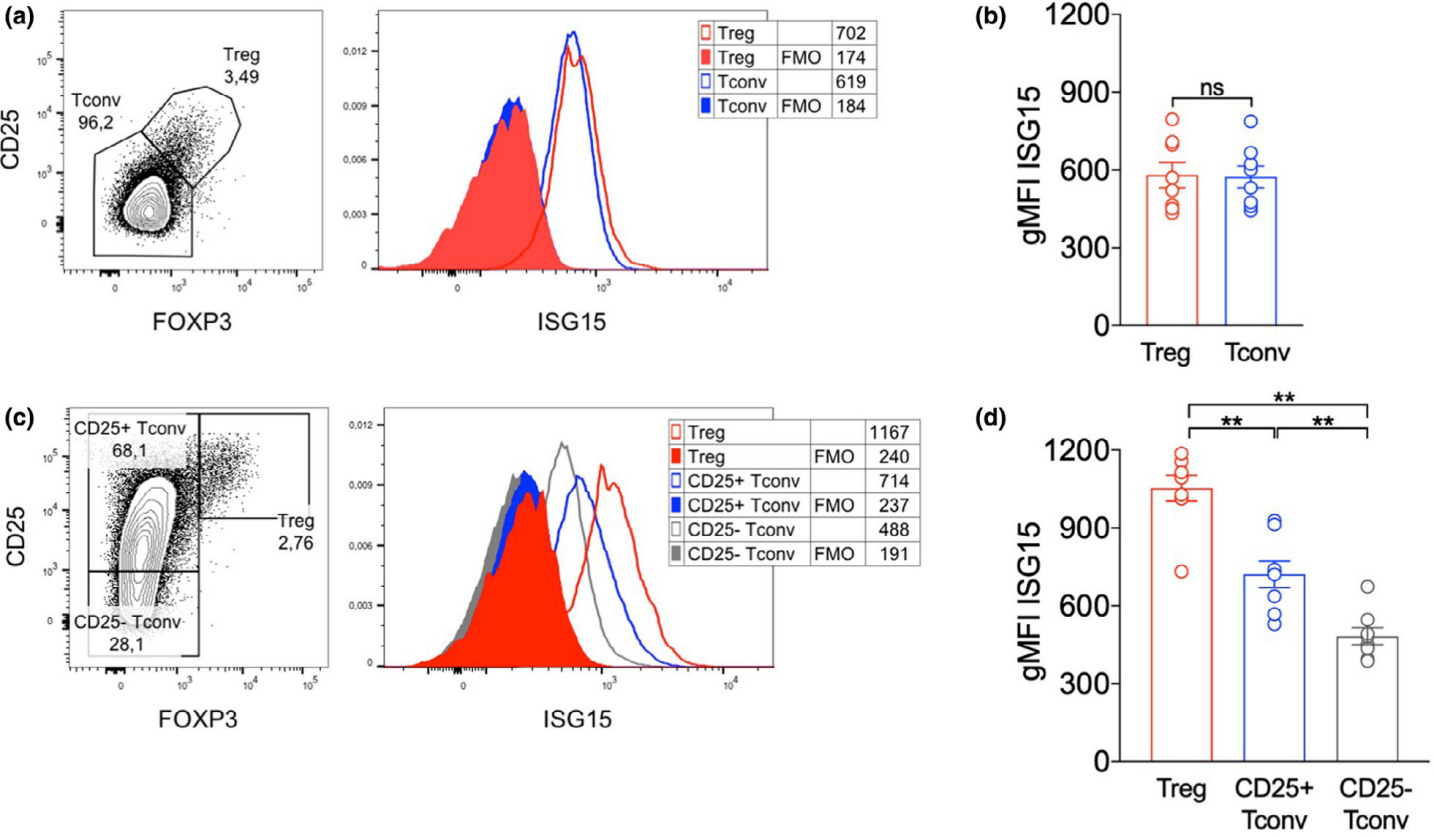
ISG15 is expressed at high levels in Tregs from HDs. **(a, b)** Peripheral blood mononuclear cells (PBMCs) were isolated from HDs, and intracellular ISG15 content was analysed *ex vivo* through intracellular flow cytometry in gated Tregs (FOXP3^+^ CD25^+^) and conventional T cells (Tconvs, FOXP3^−^ CD25^−^). Representative plots **(a)** and means ± SEM of ISG15 expression **(b)** are depicted. **(c, d)** ISG15 expression was analysed after a 48‐h culture with anti‐CD3, in gated Tregs (FOXP3^hi^ CD25^hi^), activated Tconvs (FOXP3^−^ CD25^+^) and resting Tconvs (FOXP3^−^ CD25^−^). Representative plots **(c)** and means ± SEM of ISG15 expression **(d)** are depicted. In all panels, numbers indicate the geometric mean fluorescence intensity (gMFI). FMO, fluorescence‐minus‐one control. Data shown are from eight HDs, analysed in two independent experiments. **P < 0.01, by the Wilcoxon matched‐pairs test; ns, not significant.

ISG15 can affect T‐cell functions in a cell‐intrinsic or cell‐extrinsic fashion in its secreted form.^5^ To reveal any novel Treg‐intrinsic activity, we first analysed ISG15 expression by intracellular flow cytometry: we found that ISG15 was expressed at similar levels in Tregs and conventional T cells (Tconvs), gated from PBMCs freshly obtained from healthy donors (HDs; Figure 2a and b). After a 48‐h activation *in vitro*, we could observe that activated Tregs (FOXP3^hi^ CD25^hi^) expressed ISG15 at significantly higher levels than both activated (FOXP3^−^ CD25^+^) and resting (FOXP3^−^ CD25^−^) Tconvs (Figure 2c and d). This is in line with data from the Human Cell Atlas showing higher *ISG15* mRNA expression in activated compared to resting Tregs.^18^

**Figure 3:**
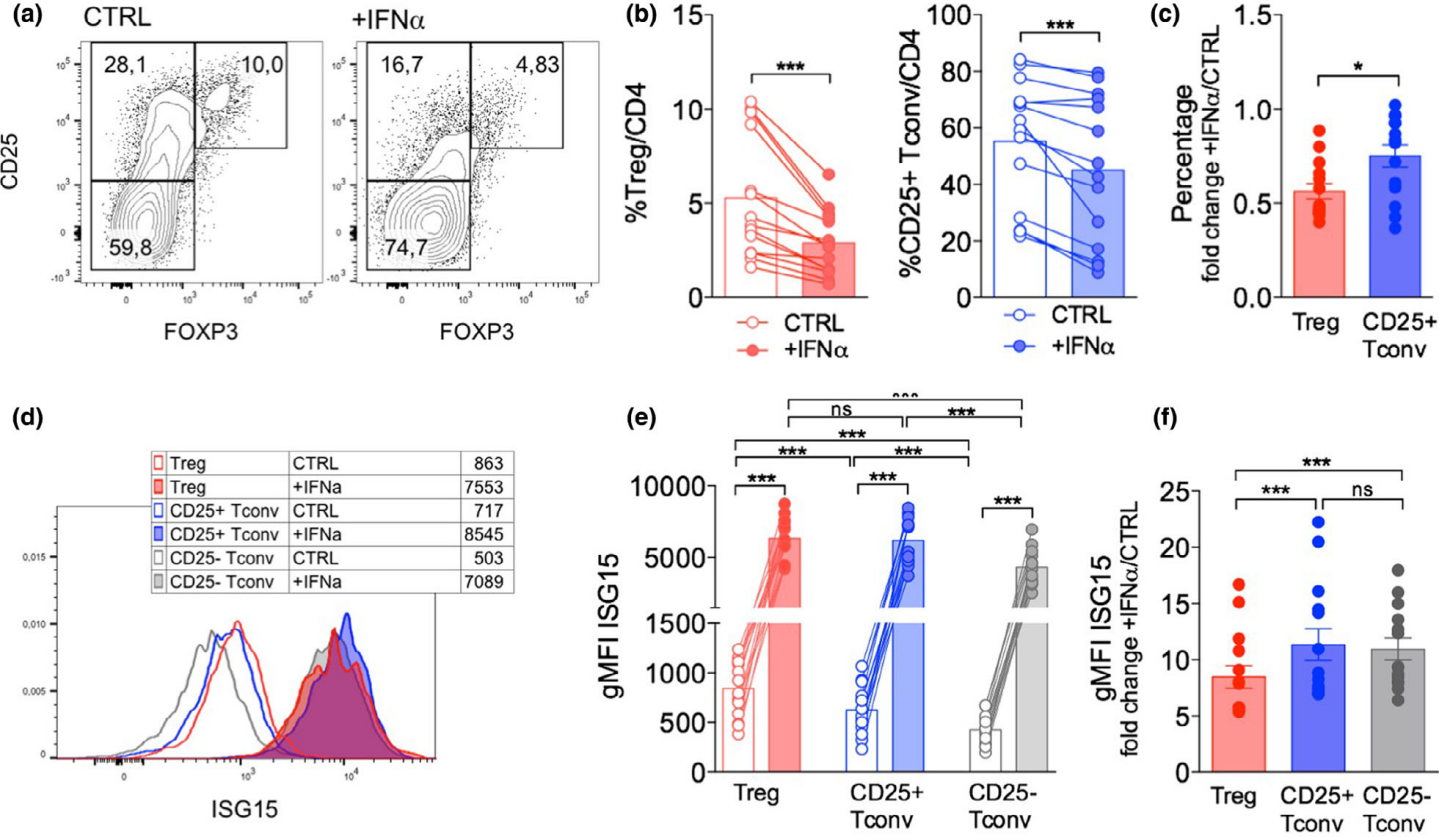
ISG15 can be induced in Tregs following IFN treatment. Peripheral blood mononuclear cells (PBMCs) were isolated from HDs and cultured 48 h with anti‐CD3 alone (CTRL) or plus recombinant human IFNα (10^4^ IU mL^−1^); then, Treg frequency and ISG15 expression were analysed by intracellular flow cytometry. **(a)** Representative contour plots and **(b)** means ± SEM of Tregs and activated (CD25^+^) Tconv percentages are shown. **(c)** Fold change in Treg and CD25^+^ Tconv frequency was calculated as the +IFNα/CTRL ratio. **(d)** Representative histograms and **(e)** means ± SEM of ISG15 expression (estimated as geometric mean fluorescence intensity, gMFI) are shown in the indicated subsets and conditions. **(f)** Fold change in ISG15 expression was calculated as the +IFNα/CTRL ratio. *P < 0.05, ***P < 0.005, by the Wilcoxon matched‐pairs test; ns, not significant.

Confirming our previous findings,^16^ the supplementation of IFNα (at 10^4^ IU mL^−1^ for 48 h) in the PBMC culture *in vitro* severely restrained T‐cell activation, reducing the percentages of both activated Tconvs and activated Tregs (Figure 3a and b). A similar (though less potent) effect was obtained adding CpG to the culture (not shown), which is known to induce type I IFN release by plasmacytoid dendritic cells.^19^ Of note, the extent of the IFNα‐induced reduction was significantly higher for activated Tregs compared to activated Tconvs (Figure 3c). Tregs, but not the other subsets, showed a significant decline of their absolute counts in culture (Supplementary figure 1a). The estimation of cycling (Ki67^+^) or apoptotic (AnnexinV^+^) cell frequency by flow cytometry revealed that Tregs contained the highest proportion of cycling cells and that proliferation (but not apoptosis) was more affected by IFNα exposure (Supplementary figure 1b and c). Overall, these data speak in favor of the preferential sensitivity of Tregs to IFN effects, and in particular with its anti‐proliferative (more than its pro‐apoptotic) functions, in line with what we observed *in vivo* in resting state in ISG15 deficiency, and in CHC patients undergoing pegIFN/ribavirin therapy.^16^

**Figure 4:**
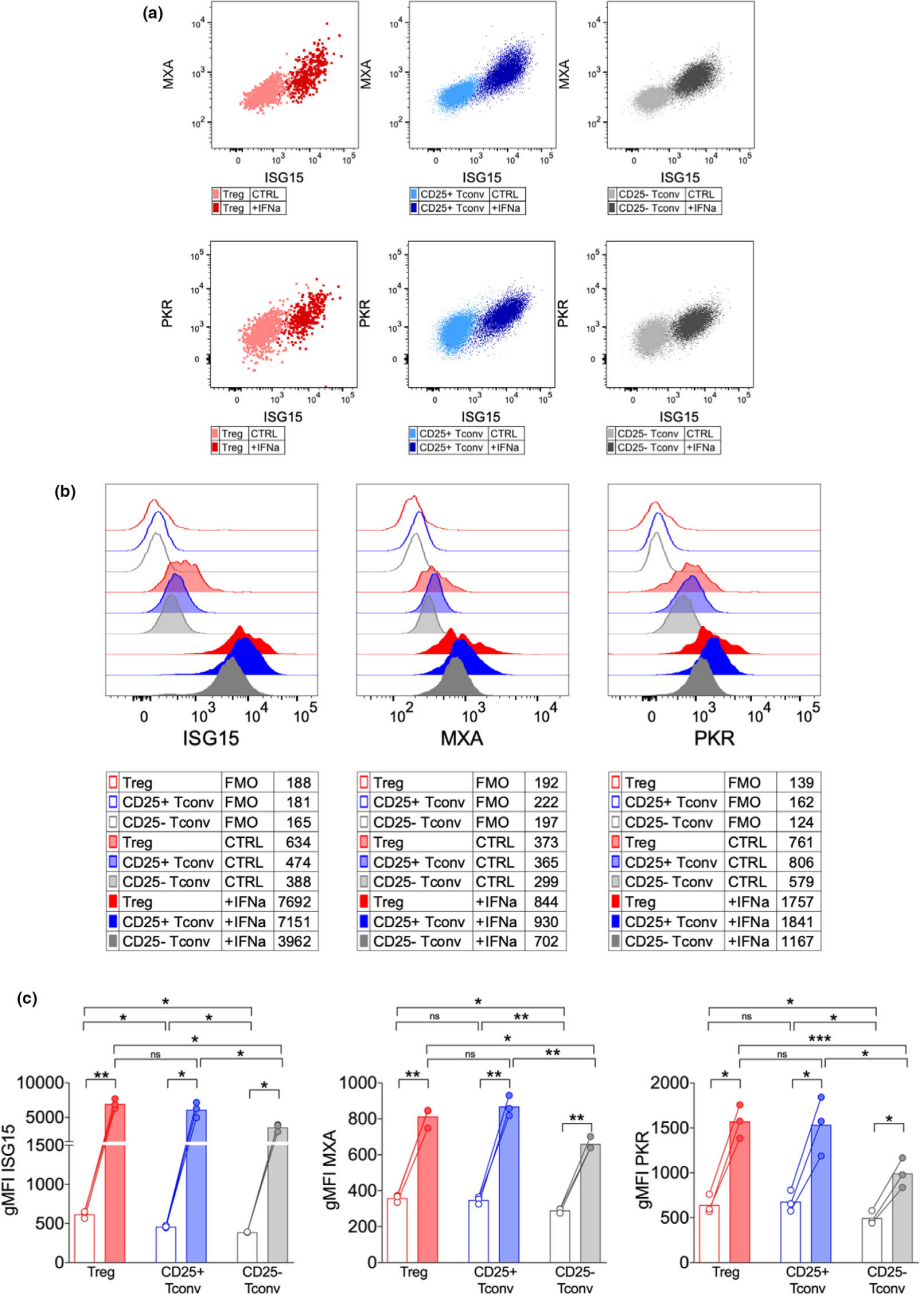
Only ISG15, but not MXA and PKR, are more expressed by activated Tregs compared to Tconvs. Peripheral blood mononuclear cells (PBMCs) were isolated from HDs (*n* = 3) and cultured 48 h with anti‐CD3 alone (CTRL) or plus recombinant human IFNα (10^4^ IU mL^−1^); then, the expression of ISG15, MXA and PKR was analysed by intracellular flow cytometry. **(a)** Representative overlays in the indicated subsets/conditions, showing coexpression of ISG15 and MXA or PKR. **(b)** Representative histograms and **(c)** means ± SEM of ISG15, MXA or PKR expression (estimated as geometric mean fluorescence intensity, gMFI) are shown in the indicated subsets and conditions. FMO, fluorescence‐minus‐one control. *P < 0.05, **P < 0.01, ***P < 0.005, by the paired *t*‐test; ns, not significant.

In our *in vitro* setting, we could detect a huge increase of ISG15 protein levels in all the three tested T‐cell populations when exposed to IFNα for 48 h (Figure 3d and e). Notably, the extent of such increase was slightly but significantly lower in activated Tregs compared to activated Tconvs (Figure 3f), probably because Tregs already displayed higher ISG15 protein levels than Tconvs when activated in the absence of exogenous IFNα. The analysis of ISG15 expression in combination with MXA and PKR, through intracellular flow cytometry, allowed demonstrating that all these three ISGs are expressed by *in vitro* activated Tregs as well as Tconvs and are strongly upregulated following IFNα treatment, being co‐expressed (Figure 4a). However, only ISG15, and not MXA or PKR, was specifically higher in Tregs than in Tconvs without IFNα exposure (Figure 4b and c), denoting the peculiar link of ISG15 with Treg functions. Altogether, these findings reveal that Tregs upregulate ISG15 upon activation and even more when exposed to IFNα and underscore the possibility of a Treg‐intrinsic role for ISG15 in modulating IFNα responsiveness.

### ISG15 silencing enhances Treg contraction in response to IFNα

**Figure 5:**
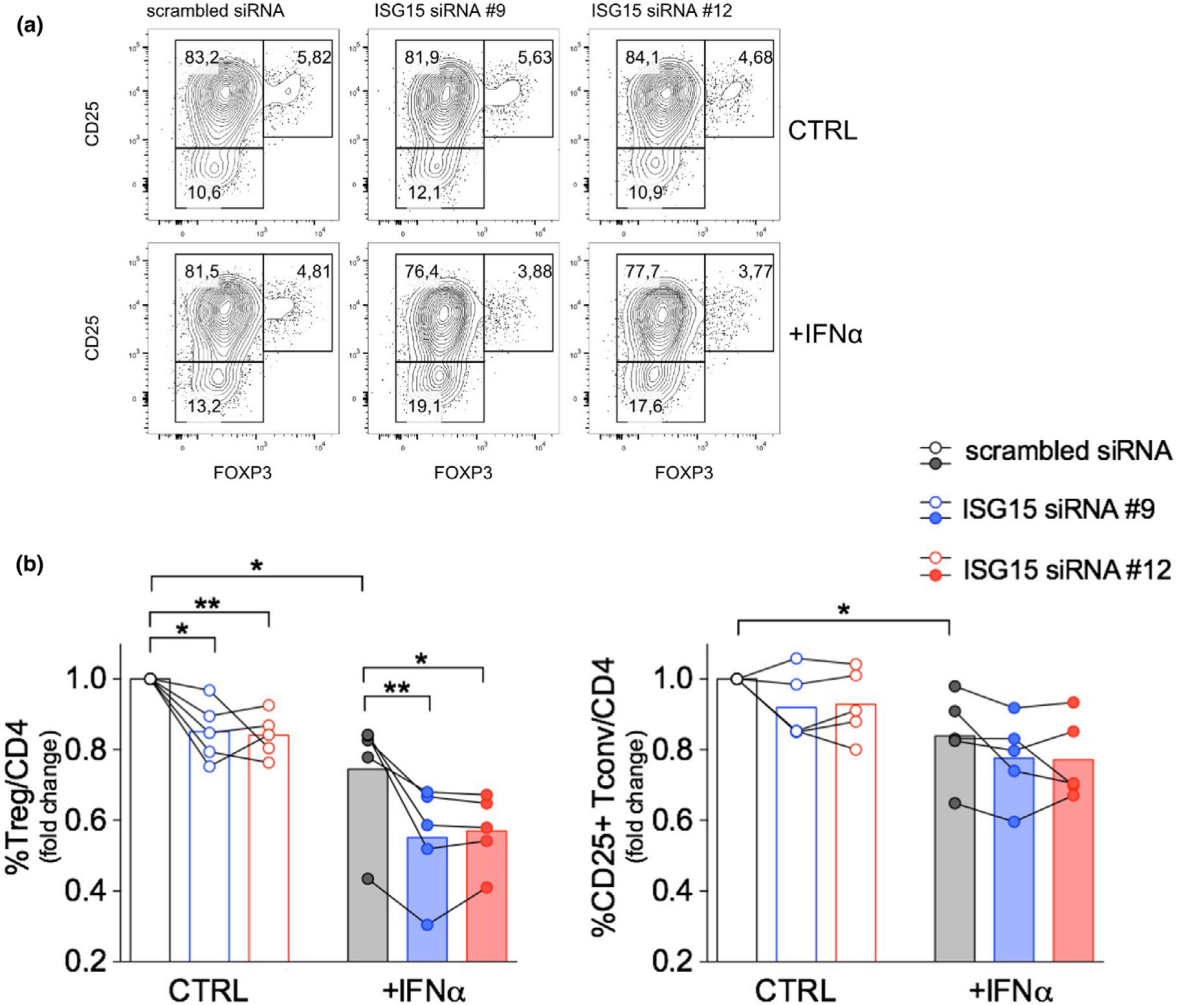
Treg‐intrinsic ISG15 protects from IFN‐induced contraction *in vitro*. Untouched CD4 T cells were isolated from peripheral blood mononuclear cells of HDs and transiently transfected with two different siRNAs targeting ISG15 (#9 or #12)^11^ or with scrambled siRNA. Cells were stimulated 18 h with or without (CTRL) recombinant human IFNα (10^2^ IU mL^−1^). Percentage of Tregs and CD25^+^ Tconvs were analysed by flow cytometry. **(a)** Contour plots from one representative experiment are shown. **(b)** Fold change of Treg and CD25^+^ Tconv percentages, relative to scrambled siRNA transfection in CTRL condition, was calculated from each donor; the bars show the results from 5 HDs independently analysed. *P < 0.05, **P < 0.01, by the paired *t*‐test.

Some data point to a role for ISG15 in blocking IFN‐STAT1 signalling pathway.^6^, ^17^ Therefore, our data led us to hypothesise that ISG15 may reduce Treg susceptibility to IFNα‐induced and STAT1‐mediated effects. To test this hypothesis, we set up an *in vitro* experiment of siRNA‐mediated ISG15 silencing. First, to avoid any confounding element coming from non‐CD4 PBMCs, we moved to a setting of untouched CD4 T‐cell isolation and culture. Second, we shortened the stimulation time to 18 h instead of 48 h, based on preliminary experiments showing disappearance of FAM‐conjugated siRNA content in cells after that time point. Third, we stimulated cells with anti‐CD3/anti‐CD28 coated beads, since soluble anti‐CD3 alone was not suitable for efficient stimulation and costimulation of isolated CD4 T cells (not shown). Fourth, we lowered the dose of IFNα from 10^4^ to 500 IU mL^−1^, to avoid profound decrease of Treg frequency and thus allow consistent analyses in residual Tregs. Indeed, we confirmed that IFNα induced Treg reduction and ISG15 upregulation also in this setting (Supplementary figure 2a). CD4 T cells were independently isolated from PBMCs of 5 HDs and electroporated with a control (scrambled) short interfering RNA (siRNA), or with two siRNAs targeting ISG15, #9 and #12, previously shown to repress *ISG15* transcripts.^11^ Viable cells were efficiently enriched after transfection with either siRNA (Supplementary figure 2b), and polyclonally stimulated *in vitro* with or without IFNα. Flow cytometry analysis of FAM‐labelled siRNA incorporation by Nucleofector™ Technology revealed a > 95% transfection efficiency with either siRNA. It also showed the decline of siRNA content already after 4 h of culture, and even more after 18 h (Supplementary figure 2c). When we analysed by intracellular flow cytometry the ISG15 protein levels, we could observe a tiny reduction in ISG15‐targeted cells, compared to scrambled siRNA‐transfected controls, only in conditions of IFNα exposure (Supplementary figure 2d). This result indicated modest suppression of ISG15 protein content only under active induction. Despite such low protein repression, we could observe in all samples a significant reduction of Treg frequency when cells had been transfected with either ISG15‐targeting siRNA compared to scrambled oligo. Such reduction was detected after *in vitro* culture without exogenous IFNα and was even stronger under IFNα exposure. Notably, in line with above data showing higher ISG15 expression in activated Tregs compared to activated Tconvs (Figure 2c and d), transfection with ISG15‐targeting siRNAs did not significantly impact the frequency of activated Tconvs (Figure 5a and b). These results functionally demonstrate that ISG15 protects Tregs from IFNα‐induced contraction *in vitro*. Based on our previous publication,^16^ on existing literature,^20^ and on the above data, we hypothesise that reduced activation, more than increased cell death, concur to such contraction and thus could be antagonised by ISG15.

Human ISG15 deficiency is characterised by systemic type I‐IFN‐mediated inflammation.^12^ To investigate the hypothesis that ISG15 also plays a role in IFN response by Tregs *in vivo*, we first analysed the frequencies of circulating Tregs in these patients. CyTOF analyses revealed no appreciable difference in Treg frequencies when compared to healthy controls at steady state (Supplementary figure 3). To assess transcriptional differences of Tregs in patients with ISG15 deficiency, we analysed a previously published single‐cell RNA‐sequencing (scRNA‐seq) data set of PBMCs.^12^

**Figure 6:**
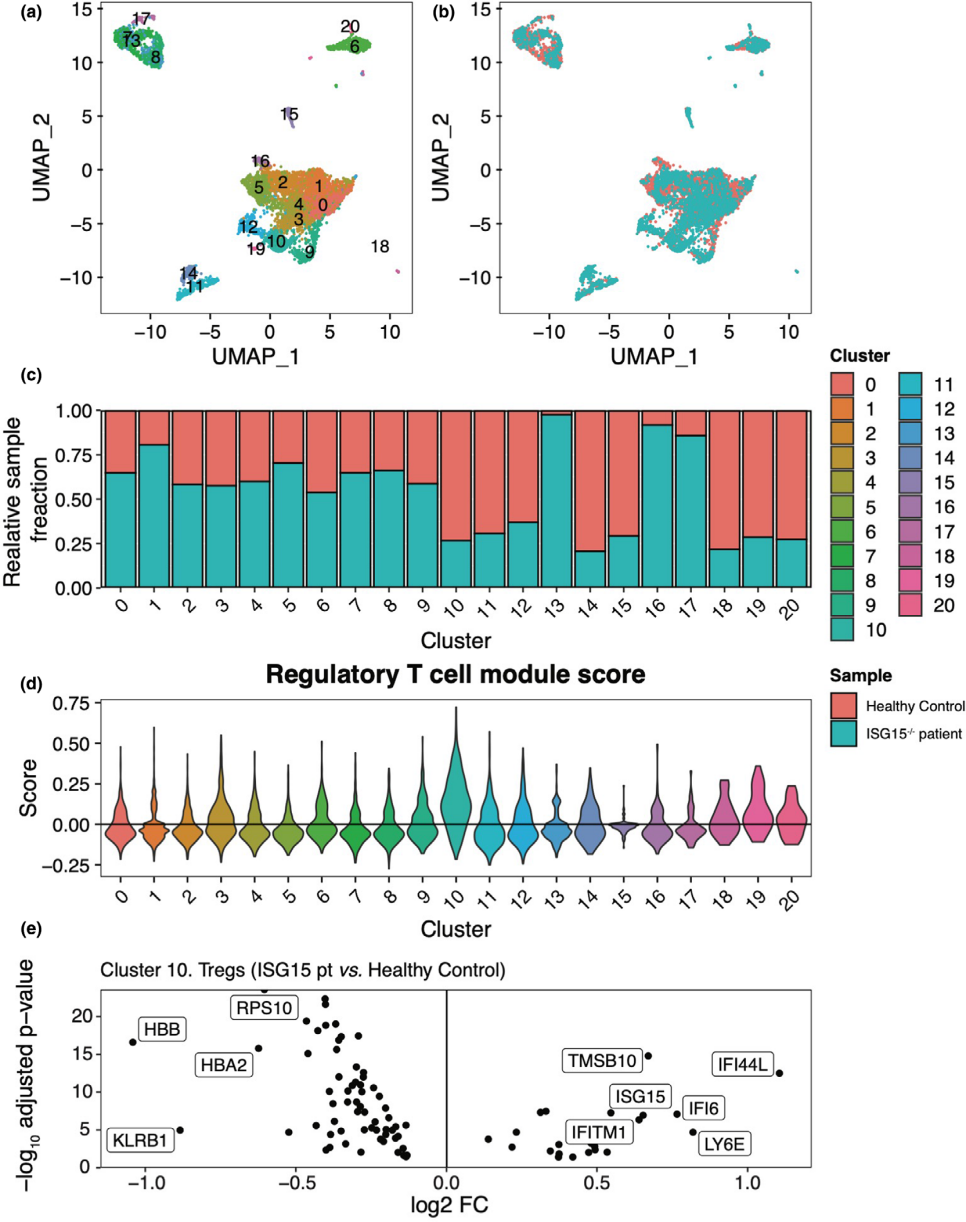
Single‐cell RNA‐sequencing analysis of ISG15^−/−^ peripheral blood mononuclear cells (PBMCs) hints at an ISG signature in regulatory T cells. **(a)** UMAP visualisation of patient and control PBMCs (*n* = 7590 cells). Points (cells) are coloured by cluster assignment. **(b)** Corresponding UMAP visualisation coloured by sample identity (Healthy control, ISG15^−/−^ patient). **(c)** Relative sample fractions of all clusters. **(d)** Regulatory T‐cell marker gene module scores across all clusters. Gene set includes *CD4*, *IL2RA* (CD25), *FOXP3*, *CD5*, *ENTPD1*, *ITGAE*, *LAG3*, *CTLA4*, *TNFRSF4* (OX40) and *TNFRSF18* (GITR). Scores represent the average expression level of the gene set subtracted by the aggregated expression of a random control feature set (*n* = 5 genes). **(e)** Volcano plot showing fold changes for genes differentially expressed between regulatory T cells (cluster 10) from the ISG15^−/−^ patient and regulatory T cells from the healthy control. Genes with a fold change greater than ± 1.5 and an adjusted *P*‐value < 0.05 are labelled.

This analysis was performed on a patient harbouring a compound heterozygous variant that did not affect *ISG15* mRNA but reduced/abolished protein levels.^12^ Unsupervised graph‐based clustering of 7, 590 PBMCs resolved 20 clusters, representing cell types present in the major lymphoid and myeloid immune lineages (Figure 6a). All clusters were represented in PBMCs from a patient with ISG15 deficiency (*N* = 1) and a healthy control (*N* = 1; Figure 6b and c). Next, we calculated a Treg module score (calculated by assessing the expression of manually curated Treg gene set) at a single‐cell resolution identifying cluster 10 as Tregs (Figure 6d). Differential gene expression suggests an elevated type I IFN gene signature in Tregs from the ISG15 deficient patient, relative to Tregs from the healthy control (Figure 6e). These data pose that, in the context of human ISG15 deficiency, Tregs also display a higher IFN response at the basal state, corroborating the notion that ISG15 also plays a role in Tregs to protecting from exaggerated type‐I IFN signals.

### High ISG15 expression correlates with lower IFN‐STAT1 signals and higher Treg frequency in SLE patients

**Figure 7:**
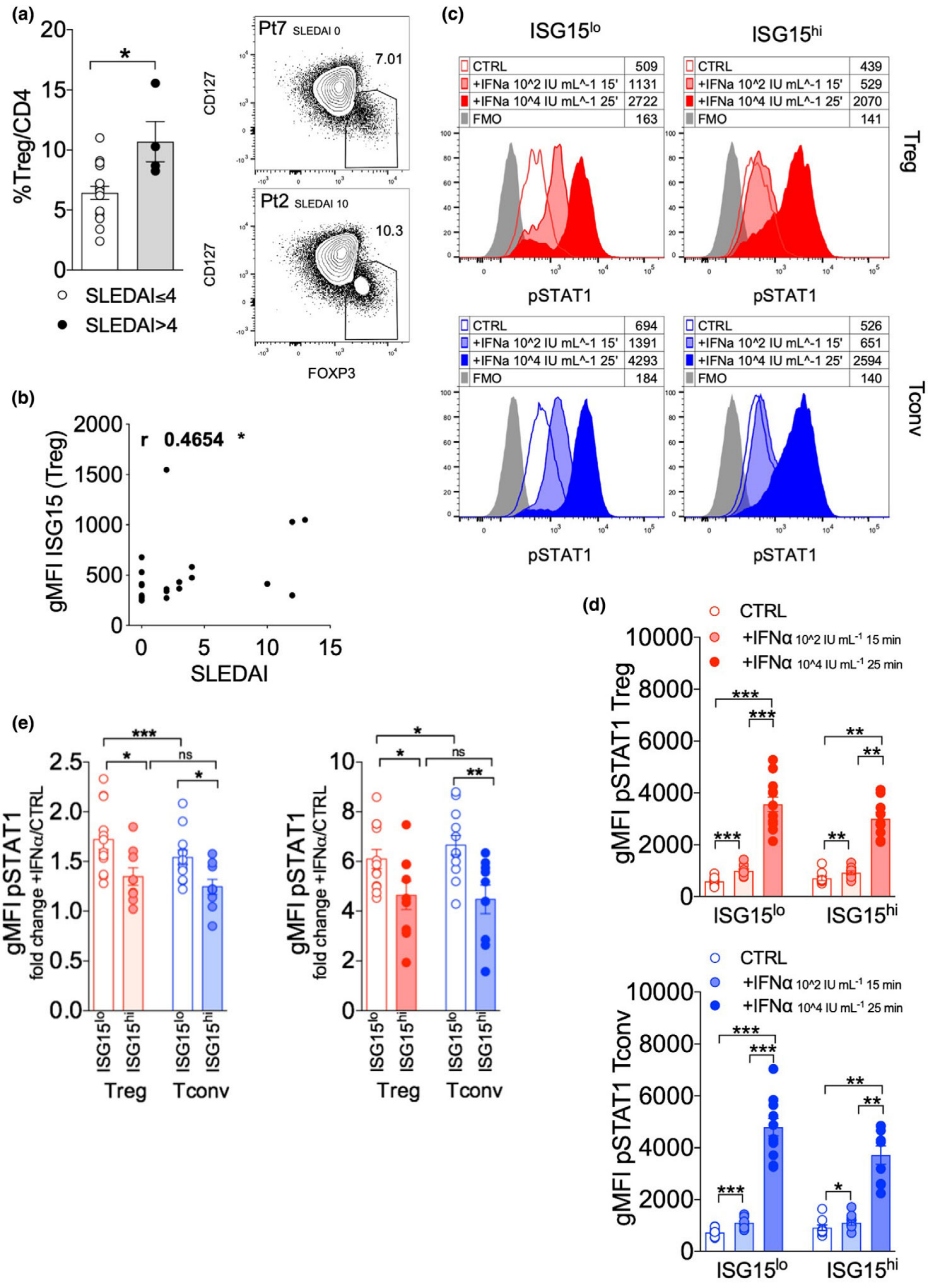
ISG15 hinders STAT1 phosphorylation of Tregs from systemic lupus erythematosus (SLE) patients exposed to IFN ex vivo. **(a)** Treg frequency (as FOXP3^+^ CD127^lo^ cells) and ISG15 expression in Tregs were estimated through flow cytometry in peripheral blood mononuclear cells (PBMCs) obtained from SLE patients (*n* = 21), stratified according to their SLEDAI score into ≤ 4 (*n* = 17) or > 4 (*n* = 4). Dot plots from two representative patients, Pt7 and Pt2, are shown. *P < 0.05, by the Mann–Whitney *U‐*test. **(b)** Spearman correlation between the SLEDAI score and the gMFI of ISG15 in Tregs in all patients. *P < 0.05. **(c–e)** SLE patients were stratified into ISG15^lo^ (*n* = 12) and ISG15^hi^ (*n* = 9), and intracellular staining for pSTAT1 was performed in gated Tregs and Tconvs from PBMCs, either freshly isolated (CTRL) or pulsed *ex vivo* with recombinant human IFNα (10^2^ IU mL^−1^ for 15 min or 10^4^ IU mL^−1^ for 25 min). **(c)** Representative histograms overlay and **(d)** means ± SEM of pSTAT1 levels (estimated as geometric mean fluorescence intensity, gMFI) are shown in the indicated subsets and conditions. **(e)** Fold change in pSTAT1 levels was calculated as the ratio between IFN stimulation at 10^2^ IU mL^−1^ for 15 min and CTRL (left plot), or between IFN stimulation at 10^4^ IU mL^−1^ for 25 min and CTRL (right plot). *P < 0.05, **P < 0.01, ***P < 0.005, by the Mann–Whitney *U‐*test between ISG15^lo^ and ISG15^hi^ patients, and by the Wilcoxon matched‐pairs test between Tregs and Tconvs or between CTRL and +IFNα; ns, not significant.

The data above suggest that ISG15 may act as a Treg‐protective molecule under pharmacological IFNα exposure. Thus, we tested whether ISG15 played this role also in those autoimmune diseases where type I IFNs are produced endogenously and contribute to immunopathogenesis, such as SLE.^4^ Interestingly, a certain fraction of IFN‐treated patients develop autoimmune disorders including a SLE‐like disease.^21^ Contradictory findings are present in the literature regarding Treg percentages and functions in SLE patients, which is likely to be due to the clinical and immunological heterogeneity of this disease.^22^, ^23^ We analysed Tregs in a cohort of SLE patients, showing variable disease activity (Table 1). In line with published data,^24^ we found increased frequency of circulating Tregs (identified as FOXP3^+^ CD127^lo^ cells) in SLE patients with active disease (SLEDAI > 4) compared to inactive disease (SLEDAI ≤ 4; Figure 7a). Patients with active disease also displayed a trend for higher expression of *ISG15*, *PKR* and *MXA* mRNAs in PBMCs (not shown), being the three genes generally coregulated, in line with data in the literature.^4^ More importantly, the SLEDAI score positively correlated with the expression of ISG15 protein in Tregs (Figure 7b): this suggested that, in active disease, the ISG15 increase that probably follows IFN exposure may favor Treg accumulation.

**Table 1:** Characteristics of systemic lupus erythematosus (SLE) patients

Pt no.	Age (years)	Sex (M/F)	Disease duration (months)	SLEDAI	ISG15 mRNA	ISG15 ^hi^ or ^lo^
1	29	F	131	12	0.063	ISG15^lo^
2	49	F	304	10	0.058	ISG15^lo^
3	45	F	132	2	0.391	ISG15^hi^
4	31	F	132	13	0.241	ISG15^hi^
5	21	F	11	0	0.149	ISG15^hi^
6	45	F	95	2	0.038	ISG15^lo^
7	46	M	251	0	0.045	ISG15^lo^
8	31	F	14	12	0.378	ISG15^hi^
9	38	F	180	0	0.023	ISG15^lo^
10	36	F	240	0	0.026	ISG15^lo^
11	52	F	72	0	0.192	ISG15^hi^
12	52	F	264	4	0.142	ISG15^hi^
13	34	F	84	0	0.245	ISG15^hi^
14	52	F	180	3	0.054	ISG15^lo^
15	30	F	22	3	0.204	ISG15^hi^
16	53	F	47	2	0.039	ISG15^lo^
17	49	F	252	4	0.372	ISG15^hi^
18	59	F	384	0	0.073	ISG15^lo^
19	77	F	1	0	0.049	ISG15^lo^
20	32	F	175	0	0.037	ISG15^lo^
21	53	F	180	2	0.031	ISG15^lo^

We hypothesised that such effect might be due to ISG15‐mediated resistance to the IFN signal and STAT1 phosphorylation. To test this hypothesis, we assessed pSTAT1 by intracellular flow cytometry in Tregs and Tconvs from SLE patients, stratified according to their *ISG15* mRNA expression in PBMCs. Similar to CHC patients, *ISG15* mRNA levels were heterogeneous among SLE patients and allowed distinguishing ISG15^lo^ and ISG15^hi^ subgroups. Contrary to CHC patients, also *PKR* and *MXA* mRNA levels were significantly different in the two SLE patient subgroups (Supplementary figure 4a). Also contrary to CHC patients, we could not detect any significant difference in Treg frequency measured *ex vivo* in ISG15^lo^ and ISG15^hi^ SLE patients (Supplementary figure 4b). A significant correlation could be found between *ISG15* mRNA measured in total PBMCs, and ISG15 protein levels detected in Tregs by intracellular flow cytometry (Supplementary figure 4c), suggesting that Treg‐intrinsic ISG15 expression may mirror generalised induction in lympho‐monocytes.

We measured pSTAT1 in gated Tregs and Tconvs from ISG15^lo^ and ISG15^hi^ SLE patients, either untreated or after a pulsed exposure *ex vivo* to IFNα with two different doses and durations (10^2^ IU mL^−1^ for 15 min, or 10^4^ IU mL^−1^ for 25 min), recapitulating suboptimal or optimal stimulation of IFN receptors respectively. In both Tregs and Tconvs, the two tested conditions of IFNα exposure could induce graded STAT1 phosphorylation in samples from ISG15^lo^ as well as ISG15^hi^ SLE patients (Figure 7c–e). The basal STAT1 phosphorylation levels did not significantly differ between ISG15^lo^ and ISG15^hi^ patients in either Tregs or Tconvs. However, looking more carefully at pSTAT1 fold change between untreated and IFNα‐treated cells, we could reveal that Tregs and Tconvs from ISG15^lo^ patients displayed a significantly higher capability than ISG15^hi^ to phosphorylate STAT1 in response to IFNα at both doses and durations tested (Figure 7e). In more detail, Tregs from ISG15^lo^ patients were more responsive than Tconvs to the low IFNα exposure, and less responsive than Tconvs to the high IFNα exposure (Figure 7e), suggesting a differential fine sensitivity of the two subsets to the IFN‐STAT1 signal.

We then tested whether the different sensitivity of Tregs from ISG15^lo^ or ISG15^hi^ patients might impact Treg persistence when cultured *in vitro* in the presence of exogenous IFNα. While STAT1 phosphorylation was assessed at very early time points from IFNα exposure (15–25 min), Treg frequencies were evaluated after 48 h of culture, as we did for HDs, to provide enough time for detecting a relevant decrease of Treg percentage. Indeed, as we shown above, Treg decline was quite modest after only 18 h of culture. To this aim, PBMCs from ISG15^lo^ and ISG15^hi^ SLE patients were stimulated with anti‐CD3 for 48 h, with or without 10^4^ IU mL^−1^ IFNα. We found that Tregs and Tconvs from the two patient subgroups were similarly capable to upregulate ISG15 under IFNα supplementation (Supplementary figure 5a). Of note, pSTAT1 levels were high in untreated T cells and minimally increased by exogenous IFNα in this setting, probably due to the abundance of other STAT1‐stimulating cytokines released in the PBMC culture (Supplementary figure 5b).

**Figure 8:**
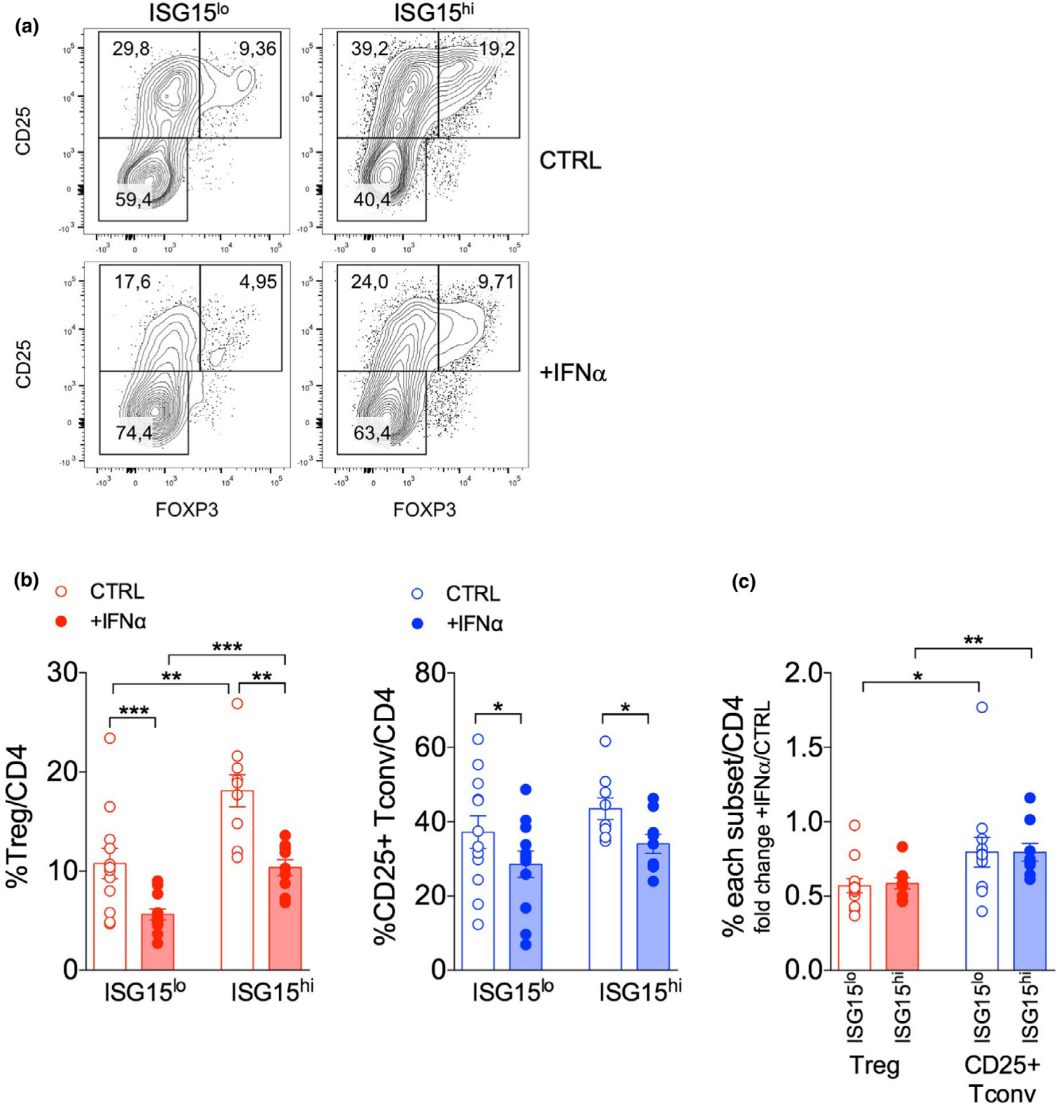
ISG15 dictates IFN sensitivity of Tregs ex vivo from systemic lupus erythematosus (SLE) patients. Peripheral blood mononuclear cells were obtained from ISG15^lo^ and ISG15^hi^ SLE patients and stimulated 48 h with anti‐CD3 alone (CTRL) or plus recombinant human IFNα (10^4^ IU mL^−1^); then, frequency of Tregs and CD25^+^ activated Tconvs was analysed by flow cytometry. **(a)** Representative contour plots and **(b)** means ± SEM of Treg and CD25^+^ Tconv percentages are shown in the indicated patient subgroups and conditions. **(c)** Fold change in Treg or CD25^+^ Tconv percentages was calculated as the +IFNα/CTRL ratio. *P < 0.05, **P < 0.01, ***P < 0.005, by the Mann–Whitney *U‐*test between ISG15^lo^ and ISG15^hi^ patients, and by the Wilcoxon matched‐pairs test between Tregs and Tconvs or between CTRL and +IFNα

We observed that IFNα was able to reduce the percentage of activated Tregs and Tconvs from ISG15^lo^ and ISG15^hi^ samples (Figure 8a and b). However, the effect was again significantly more pronounced in Tregs than in Tconvs (Figure 8c), replicating the data obtained in HDs (Figure 3c). Also in line with data in HDs, the decline of Tregs in response to IFNα was confirmed in terms of absolute cell counts, and this event was related to reduced frequency of cycling (Ki67^+^) cells rather than reduced frequency of apoptotic (AnnexinV^+^) cells (Supplementary figure 6). Strikingly, we observed that, irrespective of IFNα supplementation, the frequency of activated Tregs, but not of activated Tconvs, was consistently and significantly higher in ISG15^hi^ compared to ISG15^lo^ SLE patients (Figure 8a and b). In summary, our results demonstrate that, in SLE patients, high ISG15 levels are associated to enhanced Treg frequency after a short‐term culture of PBMCs, even under IFNα exposure.

## Discussion

Our results uncover a novel Treg‐intrinsic role for ISG15 in dampening excessive type I IFN signalling, which may protect Tregs from the effects of an acute exposure to type I IFNs occurring *in vivo* during therapy for CHC or during disease reactivation in SLE. Notably, this event may take place also during viral infections in which type I IFNs are produced, such as in mild forms of SARS‐CoV2 infection.^25^

In CHC patients, from day 0 to day 2 of pegIFN/ribavirin therapy, a higher inducibility of ISG15 (observed in patients who were classified as ISG15^lo^ at baseline) correlated with a stronger persistence of Tregs under IFN exposure *in vivo*. Even though we could not assess, in this cohort, the ISG15 protein expression in Tregs, this result led us to speculate that ISG15 may hinder the pro‐apoptotic and anti‐proliferative effects through which IFN‐STAT1 signalling restrains the Treg population.^16^ ISG15 has been shown to promote refractoriness to IFN signalling in the liver and thus to predict poor virological response of CHC patients to pegIFN/ribavirin.^7^, ^8^, ^10^ In HIV‐infected patients, only ISG15 (among the four ISGs tested) correlated positively with viraemia, supporting the view of ISG15 as a pro‐viral factor in HIV‐related disease, and was also associated with markers of immune suppression and T‐cell dysfunction.^26^ Accordingly, our previous report proposed that the anti‐inflammatory ISG15 counterbalances the pro‐inflammatory functions of other ISGs (e.g. MXA and PKR), ultimately contributing to establish a status of low‐grade/long‐lasting inflammation in patients persistently infected with HCV.^9^ In animal models of viral infection, type I IFNs play a paradoxically dual role, being able to suppress viral replication in acute infections and to promote viral persistence in chronic settings.^27^, ^28^ This paradoxical phenomenon may be attributed to the refractoriness that chronic IFN exposure establishes against acute IFN stimulation, which has been proposed to occur in chronic HCV or HIV infections,^10^, ^28^ and where ISG15 may play a key role.^8^, ^11^ Here we propose that a Treg‐intrinsic activity of ISG15 may contribute to the resistance to immunomodulatory effects of IFN.

Whether this event contributes to the clinical response to pegIFN/ribavirin therapy remains to be clarified. In mouse models of viral infection, the IFN‐driven Treg elimination allows mounting antiviral immunity and clearing the infection.^13^, ^14^ However, ISG15 may not be involved in controlling Treg dynamics in these settings, since ISG15 does not affect IFN response in murine cells.^29^ Also in a primate model of subinfectious HCV exposure, Tregs were responsible for suppressing the development of protective memory responses.^30^ We have previously shown that the decline of activated Treg frequency at day 2 of pegIFN/ribavirin therapy was stronger in CHC patients experiencing virological response.^16^ Here, we show a trend for higher rates of clinical responses in ISG15^lo^ patients, in line with the notion that increased ISG15 levels may predict refractoriness to therapeutic IFN administration.^10^ Notably, the other two tested ISGs, *PKR* and *MXA*, were not variable among patient subgroups at baseline and did not show different inducibility from day 0 to day 2 between ISG15^lo^ and ISG15^hi^ patients: this confirms that ISG15 expression may have a distinct regulation and a different meaning compared to other ISGs. Interestingly, we found that also MXA and PKR are expressed in activated Tregs and Tconvs and are strongly upregulated by IFNα, however only ISG15 showed higher expression in Tregs compared to other subsets following activation in the absence of IFNα, strengthening the idea that ISG15 may play regulatory roles differently from other ISGs.

Among CHC patients, ISG15^lo^ are those experiencing a higher ISG15 induction from day 0 to day 2 and are also more protected from Treg reduction. Therefore, while a low ISG15 basal level seems to correlate with clinical response, a high ISG15 inducibility is associated with Treg persistence. Further experiments may have been needed to demonstrate the role of Tregs in therapy response. However, antiviral agents have replaced pegIFN/ribavirin for CHC treatment in recent years, making further studies not possible.^15^ If corroborated by further analyses, baseline ISG15 expression could have been considered as a possible biomarker predicting response to IFN therapy. Another point that has not been addressed here is the impact of ISG15 expression or inducibility in Treg response to other type I (IFNβ) and type III (IFNλ) interferons: indeed, it has been shown that repeated IFN exposure failed to induce refractoriness to IFNβ or IFNλ stimulation, despite potent USP18 induction.^31^

The description of a drug‐induced SLE in CHC patients receiving IFN‐based therapy^21^ offered insights to investigate the role of IFN in the pathogenesis of the disease. Thus, SLE was chosen as a model that could reasonably confirm what was observed in pegIFN‐treated CHC patients. However, it should be taken into account that the intensity and the duration of IFN exposure might significantly differ in the two settings. This may explain the discrepant results obtained in CHC and SLE patients, where Tregs correlated with ISG15 inducibility or ISG15 baseline level, respectively. Also contrary to CHC patients, in SLE patients not only ISG15, but also MXA and PKR show variable expression among subjects, tending to be higher in active disease. IFN signature is recognised as a hallmark of SLE and generally peaks before or during disease reactivation.^4^, ^32^ Our data identify ISG15 as possible link between IFN signature, disease activity and Treg expansion: indeed, SLE patients with active disease have higher Treg percentage, and disease activity correlates with higher Treg‐intrinsic ISG15 expression. Many papers have reported defective Treg frequencies and/or functions in SLE patients, with several contradictory findings.^23^ A refined analysis of Helios^+^ Tregs, which do not produce cytokines and show epigenetic features of Treg lineage stability, has demonstrated that their frequency increases in active SLE compared to inactive disease.^24^ Our results are in line with these findings, and also suggest that ISG15 may be the missing link between disease activity and Treg persistence. Indeed, an exogenous or endogenous IFN trigger^4^ may promote disease activation on the one side, and induce IFN signature (including ISG15) on the other side; then, ISG15 induction may sustain Treg persistence in a Treg‐intrinsic fashion, through a desensitisation to the IFN‐STAT1 signal. Several data speak in favor of this model: (1) patients with active disease have higher Treg frequency and higher Treg‐intrinsic ISG15 expression; (2) CD4 T cells from ISG15^hi^ patients (stratified according to the mRNA levels in total PBMCs) are less responsive to IFN stimulation *ex vivo* in terms of STAT1 phosphorylation; (3) after a short‐term culture *in vitro*, Treg frequency is higher in ISG15^hi^ patients irrespective of IFN exposure. Interestingly, the difference in Treg percentages could not be observed *ex vivo*, when Tregs were identified as FOXP3^+^ CD127^lo^ cells, but was clearly detected after *in vitro* culture, when Tregs were identified as FOXP3^hi^ CD25^+^ cells. It is possible that Tregs transiently downregulate FOXP3 *in vivo* especially under inflammatory conditions and IL‐2 deprivation, and promptly reacquire FOXP3 expression under optimal *in vitro* culture conditions. Indeed, others have reported CD25 downregulation by Tregs in SLE patients.^33^ Our *ex vivo* estimation of Treg frequency as the FOXP3^+^ CD127^lo^ percentage should not be compromised by these limitations; however, we obtained different results when Tregs were analysed *ex vivo* or after culture *in vitro*. Our findings are in line with the idea that human ‘latent’ Tregs can be revealed after a short‐term *in vitro* exposure to IL‐2 also from patients with autoimmune diseases.^34^ Indeed, in a very similar experimental setting (with IL‐2 produced by anti‐CD3/anti‐CD28 stimulated PBMCs), we could accurately identify latent Tregs in SLE patients and could demonstrate a clear difference between ISG15^lo^ and ISG15^hi^ patients in terms of Treg percentage. Indeed, FOXP3 protein expression alone may not faithfully identify the whole population of committed Tregs displaying extensive demethylation at the FOXP3 locus.^35^, ^36^ Of note, human Helios^+^ Tregs are enriched of cells carrying demethylated FOXP3 locus,^37^ and this may explain the higher accuracy of Helios^+^ Treg detection in SLE patients reported by others.^24^, ^33^

When we added exogenous IFNα to the *in vitro* culture of PBMCs from HDs or SLE patients, we observed a lower frequency of FOXP3^hi^ CD25^+^ Tregs, and this occurred in both ISG15^lo^ and ISG15^hi^ SLE patients. When ISG15 was silenced, this effect was even more prominent, demonstrating that ISG15 counteracts IFN effects on Tregs, possibly through the regulation of USP18‐mediated control of STAT1 phosphorylation. Supporting this idea, we could verify that USP18 was expressed in both Tregs and Tconvs and was strongly induced by IFNα in a time‐ and dose‐dependent fashion, as measured by intracellular staining of the USP18 protein (not shown). We have previously shown that IFNα has direct pro‐apoptotic and anti‐proliferative activities on Tregs *in vivo* and *in vitro* ^16^: here we show that, after *in vitro* activation, Tregs are the population containing the highest proportion of cycling (Ki67^+^) cells, in line with the literature.^38^ Notably, IFNα treatment reduced this frequency in samples from both HDs and SLE patients, without affecting the percentage of apoptotic cells, and without impacting on other CD4 subsets. Also, our present results show that the extent of Treg reduction is significantly stronger than the one of activated Tconvs, in samples from HDs as well as SLE patients despite IFNα upregulated ISG15 to a similar extent in the two populations. This may be explained with the contribution of ISG15‐independent events in dictating a higher susceptibility of activated Tregs to IFN‐driven decrease. In ISG15‐silenced cells, we could detect a hypersensitivity of Tregs to IFN‐induced reduction. This finding is reminiscent of the enhanced IFN signature that is associated to clinical manifestations of multiorgan inflammation, in patients carrying spontaneous ISG15 mutations.^11^, ^12^ In those subjects, myeloid cells were the most severely affected by hyperactivity of the IFN signal^12^; however, more subtle differences in ISG expression may be present also in CD4 T cells, or in CD4 subsets. Indeed, our data demonstrate that ISG15 deficiency has Treg‐intrinsic consequences, such as high IFN response, which may in turn contribute to the establishment and the persistence of interferonopathies in these patients.

Another possible approach in support of the role of ISG15 in Treg protection would be to force, rather than silencing, its expression *in vitro*: though potentially informative, we did not pursue this strategy, since we observed high levels of endogenous ISG15 expression especially in Tregs activated *in vitro* in the presence of IFNα, which could not be easily enhanced further. Beside any possible *in vitro* manipulation, our data *ex vivo* from SLE patients allowed assessing the impact of high or low ISG15 levels that occurred naturally *in vivo* in patients, rather than after forcing or silencing its expression *in vitro*.

In conclusion, our data support the notion that, following an inflammatory trigger and the activation of IFN response, the induction of ISG15 in Tregs may create a status of IFN refractoriness that protects these cells from excessive reduction, and thus preserve immune homeostasis. This model may explain Treg accumulation in active SLE, where a parallel expansion of Tregs may control the excessive autoimmunity. In addition, this model may also apply to other contexts of Treg expansion such as the tumor microenvironment, where IFN signature is related to efficient anti‐tumor responses^39^, and IFN refractoriness could lead to Treg accumulation. Of note, several data in the literature suggest that ISG15 may play multiple pro‐tumor roles^40^: whether these involve Treg expansion remain to be assessed. Supporting a role for Treg‐intrinsic roles of ISG15 in cancer, recent data have highlighted that intratumor Tregs express high levels of different IFN‐related genes, including ISG15.^41^

## Methods

### Patients and samples

Analyses were performed on PB samples from 15 CHC patients belonging to a previously described cohort, in which PB was collected at day 0 (before therapy) and at day 2 of therapy with pegIFN/ribavirin.^16^ PB was obtained from 21 SLE patients, whose clinical characteristics are described in Table 1. Buffy coats, anonymously provided by the Immunohematology and Transfusion Center of Policlinico Umberto I, were used to obtain PB from HDs. PBMCs were isolated by density gradient centrifugation through Lympholyte (Cedarlane, Burlington, Canada) and collected in complete RPMI 1640 Dutch modified medium containing 10% FBS (HyClone GE Healthcare Life Sciences, Chicago, IL, USA); 2 mm l‐glutamine (Sigma‐Aldrich, Saint‐Louis, MO, USA); penicillin/streptomycin, nonessential amino acids, sodium pyruvate (all from EuroClone, Pero, Italy); and 50 μm 2‐mercaptoethanol (Sigma‐Aldrich).

Human studies were performed in accordance with the ethical guidelines of the 1975 Declaration of Helsinki and approved by the Institutional Ethical Committee (Prot. 120/16). Informed consent was obtained from all patients.

The ISG15 deficient patient was enrolled in a NIAID IRB‐approved protocol and provided written informed consent for study participation in accordance with the Helsinki Declaration. The study of human subjects was also approved by the IRB of Icahn School of Medicine (New York, USA; IF2349568).

### ISG expression analysis

RNA was isolated from the PBMCs of CHC and SLE patients following a procedure already described.^16^ DNase‐I‐treated total RNA was purified using the RNeasy Mini Kit (Qiagen, Hilden, Germany), in accordance with the manufacturer's instructions. Reverse transcription was primed with oligo (dT) and random hexamers and performed with murine leukaemia virus RT (Thermo Fisher Scientific, Waltham, MA, USA). Quantitative PCR assays for *GAPDH*, *MXA*, *PKR*, and *ISG15* were done in triplicate using the LightCycler FastStart DNA SYBR Green I Master Mix in the presence of 3 mm MgCl_2_ on a LightCycler instrument (Roche, Basel, Switzerland). Sample values were normalised by calculating the relative quantity of each mRNA to that of *GAPDH* using the formula 2^−ΔCt^. The primer pairs are listed in Supplementary table 1.

### Flow cytometry

Treg frequency was assessed in the PB of CHC or SLE patients as detailed previously^16^: briefly, after dead cell exclusion through staining with Fixable Viability Dye eFluor 780 (eBioscience Thermo Fisher Scientific), cells were stained for surface markers and incubated for 20 min at 4°C with a cocktail of CD4 BV510 (BioLegend, San Diego, CA, USA) or PerCP‐Cy5.5 (BD Biosciences, San Jose, CA, USA), CD127 PE‐Cy7 (eBioscience Thermo Fisher Scientific), CD25 APC (BioLegend) or BV421 (BD Biosciences), CD14 APC‐Cy7 (BioLegend) in PBS 2% FBS; then, intracellular staining with FOXP3 PerCP‐Cy5.5 or AF700 (eBioscience Thermo Fisher Scientific), ISG15 PE (R&D Systems, Minneapolis, MN, USA), PKR AF647, MXA AF488 (Abcam, Cambridge, UK), Ki67 AF700 (BD Biosciences) was performed for 30 min at room temperature after fixation and permeabilisation with the FOXP3/Transcription Factor Staining Buffer Set according to the manufacturer's instructions (eBioscience Thermo Fisher Scientific). For AnnexinV staining, cells were stained with AnnexinV FITC (BioLegend) in AnnexinV binding buffer before surface staining according to the manufacturer's instructions. Combined intracellular staining for FOXP3, ISG15 and pSTAT1 was performed using the following protocol: (1) fixation with prewarmed Fixation/Permeabilization buffer (eBioscience Thermo Fisher Scientific) for 10 min at 37°C; (2) permeabilisation with precooled Perm Buffer III (BD Biosciences) for 30 min on ice; (3) surface and intracellular staining performed for 30 min at room temperature with a mix of ISG15 PE (R&D Systems), CD4 PerCP‐Cy5.5 (clone RPA‐T4; BD Biosciences), CD25 BV421 (BD Biosciences), FOXP3 AF700 (eBioscience Thermo Fisher Scientific) and pSTAT1 AF488 (BD Biosciences) dissolved in PBS with 0.5% BSA. Cells were washed extensively between steps, especially after Perm Buffer III incubation. Samples were analysed directly *ex vivo* or following a 48‐h culture in complete medium at 37°C, in the presence of anti‐CD3 (1 μg mL^−1^), with or without 10^4^ IU mL^−1^ recombinant IFNα (rhIFN‐α2b, Miltenyi Biotec, Bergisch Gladbach, Germany). For *ex vivo* analysis of ISG15 and pSTAT1 induction, cells were treated with recombinant IFNα in different conditions: 10^2^ IU mL^−1^ for 15 min or 10^4^ IU mL^−1^ for 25 min; after incubation at 37°C, cells were fixed without washing. Samples were acquired on the BD LSRFortessa cell analyzer (BD Biosciences) and analysed with FlowJo software, version 10.0.8r1 (Treestar, Ashland, OR, USA).

### ISG15 silencing *in vitro*

Untouched CD4 T cells were isolated from PBMCs of HDs through immunomagnetic separation with CD4^+^T‐cell isolation kit (Miltenyi Biotec) according to manufacturer's instructions. Cells were resuspended at room temperature in Amaxa Nucleofector solution (Lonza, Basel, Switzerland), mixed with a solution of 500 pmol FAM‐labelled oligomers, transferred into cuvettes and nucleofected by performing V‐024 program in Amaxa Nucleofector 2b device (Lonza). The following oligomers were used, based on previous literature^11^: ISG15‐siRNA #9 (5′‐GGA CAA AUG CGA CGA ACC U‐3′) and #12 (5′‐GCA ACG AAU UCC AGG UGU C‐3′), and a scrambled siRNA as non‐targeting control (5′‐GCC GAU CGU CGA GAC UAA U‐3′). After nucleofection, cells were collected in prewarmed complete medium. Dead cells were removed from samples with Dead cell removal kit (Miltenyi Biotec) and the recovered cells were cultured with Dynabeads (Gibco Thermo Fisher Scientific) at a 1:2 bead‐to‐cell ratio, in presence or absence of 500 IU mL^−1^ recombinant IFNα for 18 h.

### Mass cytometry

Peripheral blood mononuclear cells from an ISG15 deficient patient and eight healthy controls were stained and analysed by mass cytometry (CyTOF) at the Human Immune Monitoring Center of the Icahn School of Medicine at Mt. Sinai. Cells were barcoded, combined and then stained by incubation with antibodies against selected surface markers for 30 min on ice. Cells were then washed and fixed, resuspended in diH2O containing EQ Four Element Calibration Beads (Fluidigm, San Francisco, CA, USA) and acquired on a CyTOF2 mass cytometer (Fluidigm). Data files were normalised with a bead‐based normalisation algorithm (CyTOF software; Fluidigm) and debarcoded with CD45 gating.

### Single‐cell RNA‐seq analysis

Previously published single‐cell RNA‐sequencing data of PBMCs from a patient with human ISG15 deficiency (*N* = 1) and a healthy control (*N* = 1) from Martin‐Fernandez *et al*.^12^ was accessed and analysed. Raw gene‐cell matrices were read into the R (v4.0.1) statistical environment and analysed using Seurat (v3.2.2) for quality control, integration, clustering and differential gene expression. For quality control, data were filtered to exclude genes detected in less than three cells (per subject), to exclude cells with < 200 expressed genes (empty droplets) and to exclude cells with > 7.5% UMIs assigned to mitochondrial genes (dying cells). Filtered data were independently normalised using Seurat's SCTransform function (developer's default parameters).^12^ To account for subject‐specific effects, both data sets were integrated using Seurat's FindIntegrationAnchors and IntegrateData functions (developer's default parameters). Dimensional reduction of the integrated data set was performed by principal component analysis (PCA) and first 25 principal components were used for unsupervised graph‐based clusterings (resolution: 1.2) and visualised by Uniform manifold approximation and projection (UMAP; parameters: n.dims: 25, n.neighbors: 30, metric: cosine). Regulatory T‐cell gene signature scores were computed at single‐cell resolution using Seurat's AddModuleScore function. T regulatory marker genes included in input set were *CD4*, *IL2RA* (CD25), *FOXP3, CD5*, *ENTPD1*, *ITGAE*, *LAG3*, *CTLA4*, *TNFRSF4* (OX40) and *TNFRSF18* (GITR). Differential gene expression was conducted using Seurat's FindMarkers function using a log_2_FC threshold of 0.1 and an adjusted *P*‐value of 0.05.

### Statistical analysis

Statistical analysis was performed using Prism software (version 6; GraphPad, La Jolla, CA, USA). The Wilcoxon matched‐pairs test (two‐tailed) was applied to compare matched subpopulations/samples from the same patients, whereas the Mann–Whitney *U*‐test (two‐tailed) was used to compare independent patient subgroups. Significant outliers were identified and excluded through Grubbs' test (alpha = 0.05). Correlations were calculated using the nonparametric Spearman's correlation test (two‐tailed). Every *in vitro* assay was performed in duplicate or triplicate whenever possible. In all graphs, bars show mean ± SEM. In all tests, *P* < 0.05 was considered statistically significant.

## Author Contributions

**Ilenia Pacella:** Conceptualization; Data curation; Investigation; Methodology; Writing‐original draft. **Francesca Romana Spinelli:** Conceptualization; Data curation; Resources; Writing‐original draft. **Martina Severa:** Conceptualization; Data curation; Resources; Writing‐original draft. **Eleonora Timperi:** Methodology. **Gloria Tucci:** Methodology. **Marta Zagaglioni:** Methodology. **Fulvia Ceccarelli:** Resources. **Fabiana Rizzo:** Methodology. **Eliana Coccia:** Resources; Supervision; Writing‐original draft. **Roosheel S. Patel:** Data curation; Investigation. **Marta Martin‐Fernandez:** Data curation; Investigation; Visualization. **Dusan Bogunovic:** Conceptualization; Funding acquisition; Resources; Supervision; Writing‐review & editing. **Fabrizio Conti:** Resources; Supervision. **Vincenzo Barnaba:** Resources; Writing‐original draft. **Silvia Piconese:** Conceptualization; Data curation; Funding acquisition; Supervision; Writing‐original draft.

## Conflict of Interest

The authors have no conflict of interest to disclose.
