# Supplementary material for: ISG15 protects human Tregs from interferon alpha‐induced contraction in a cell‐intrinsic fashion
Source: Clin Transl Immunology. 2020 Dec 23;9(12):e1221. doi: 10.1002/cti2.1221 (PMC7758615; doi:10.1002/cti2.1221)
Supplement: Supplementary file 1 [file CTI2-9-e1221-s001.docx]

**ISG15 protects human Tregs from interferon alpha-induced contraction in a cell-intrinsic fashion.**

Ilenia Pacella *et al*.

**Supplementary data**

Supplementary table I. Primer pairs used for RT-PCR.

| **Gene** | **Primer** | **Sequence** |
| --- | --- | --- |
| MXA | forward | 5’-ATCCTGGGATTTTGGGGCTT-3’ |
| MXA | reverse | 5’-CCGCTTGTCGCTGGTGTC-3’ |
| PKR | forward | 5’-GGAAAGCGAACAAGGAGTAAG-3’ |
| PKR | reverse | 5’-GAATTAGCCCCAAAGCGTAG-3’ |
| ISG15 | forward | 5’-GGTGGACAAATGCGACGAA-3’ |
| ISG15 | reverse | 5’-TGCTGCGGCCCTTGTTAT-3’ |
| GAPDH | forward | 5’-ACAGTCCATGCCATCACTGCC-3’ |
| GAPDH | reverse | 5’-GCCTGCTTCACCACCTTCTTG-3’ |


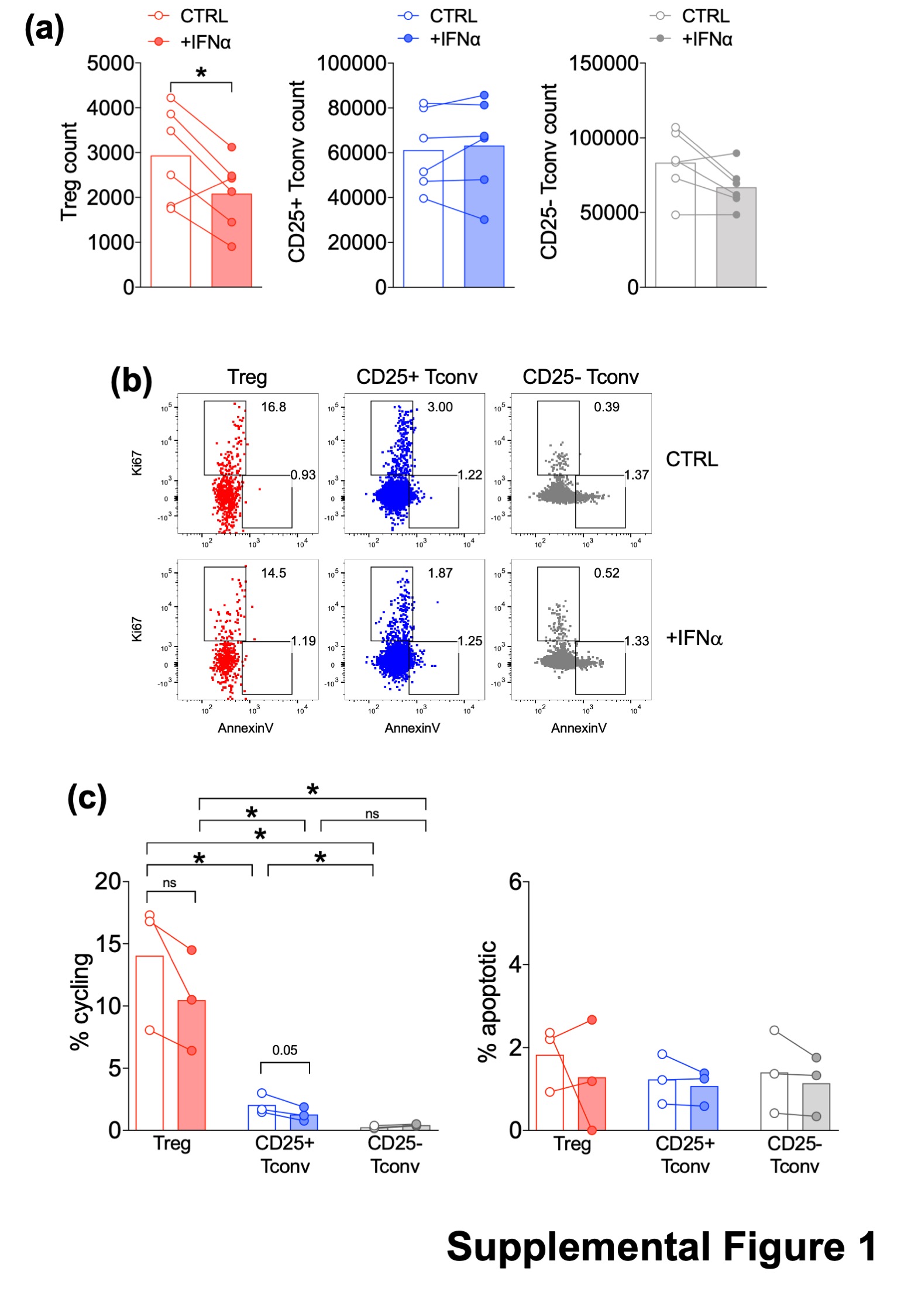


**Supplementary figure 1. IFNα exerts an antiproliferative effects on Tregs *in vitro.***

**(a)** PBMCs were isolated from HDs (n = 6) and cultured 48 hours with anti-CD3 alone (CTRL) or plus recombinant human IFNα (10^4 IU mL^-1^), then the recovered cells were manually counted. The frequencies of the indicated subsets in gated live single lymphocytes were analyzed by flow cytometry and their absolute counts were then calculated. **(b)** Representative dot plots and **(c)** means ± SEM of the percentages of Ki67+ (cycling) and AnnexinV+ (apoptotic) cells in gated Tregs, activated (CD25^+^) Tconvs and resting (CD25-) Tconvs are shown. * *P* < 0.05, by paired *t*-tests; ns, not significant.

**
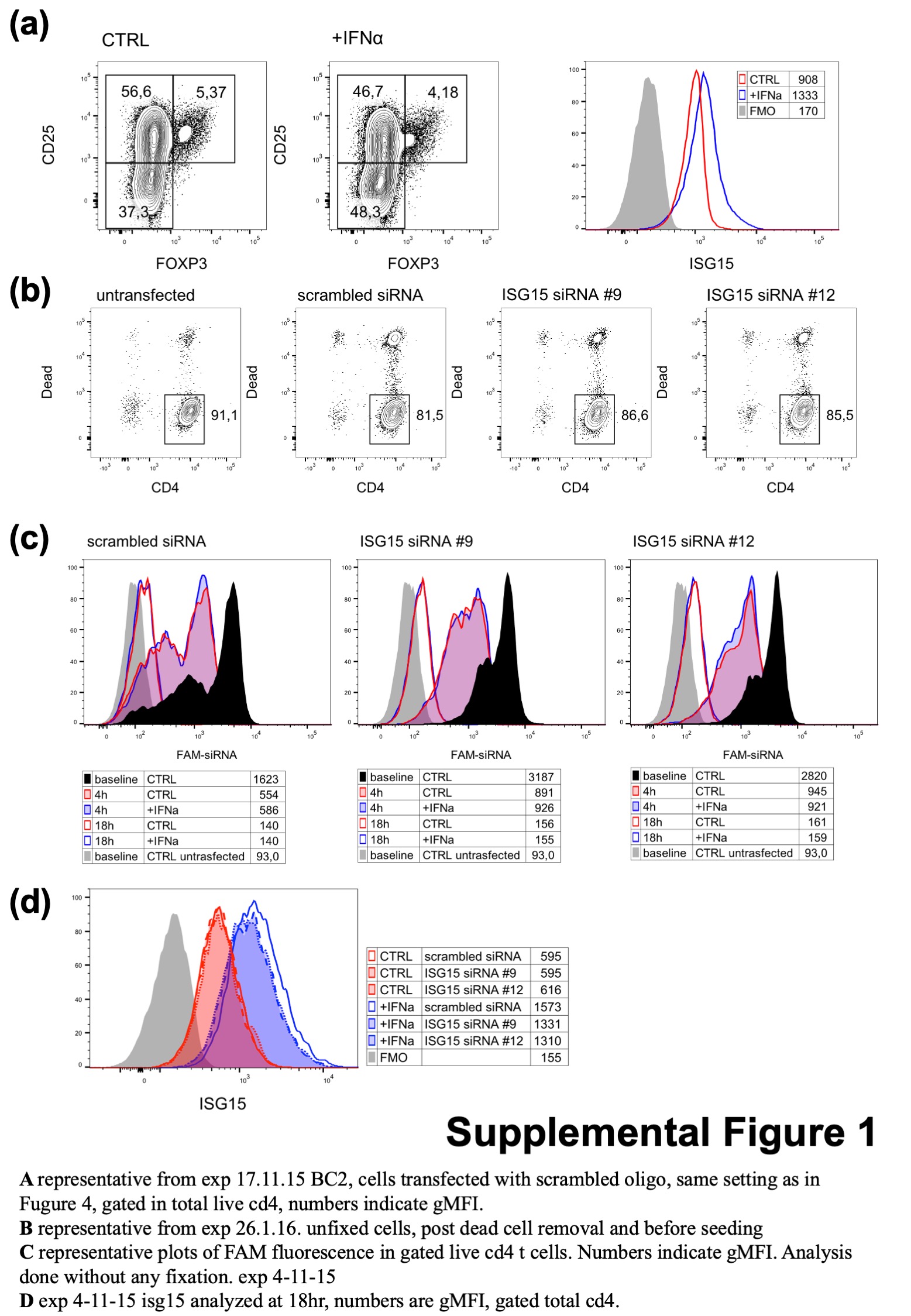
**

**Supplementary figure 2. Experimental conditions for siRNA mediated ISG15 silencing.**

**(a)** Untouched CD4 T cells were isolated from PBMCs of HDs and stimulated *in vitro* with anti-CD3/anti-CD28-coated microbeads for 18 hours, with or without (CTRL) recombinant human IFNα (10^2 IU mL^-1^). Percentage of Tregs and CD25^+^ Tconvs, and intracellular ISG15 protein content were analyzed by flow cytometry. **(b)** Isolated CD4 T cells were transfected with two different siRNAs targeting ISG15 (#9 or #12) or with scrambled siRNA, then live cells were immunomagnetically enriched, and purity and viability were checked by flow cytometry. **(c)** After transfection, CD4 T cells were stimulated with or without IFNα for 4 or 18 hours, and then FAM-oligo content was assessed by flow cytometry in gated live CD4 T cells from unfixed samples. **(d)** The intracellular protein level of ISG15 was analyzed by flow cytometry in gated live CD4 T cells after 18 hours of culture in the indicated conditions. In all plots, numbers indicate the gMFI. FMO, fluorescence-minus-one control.


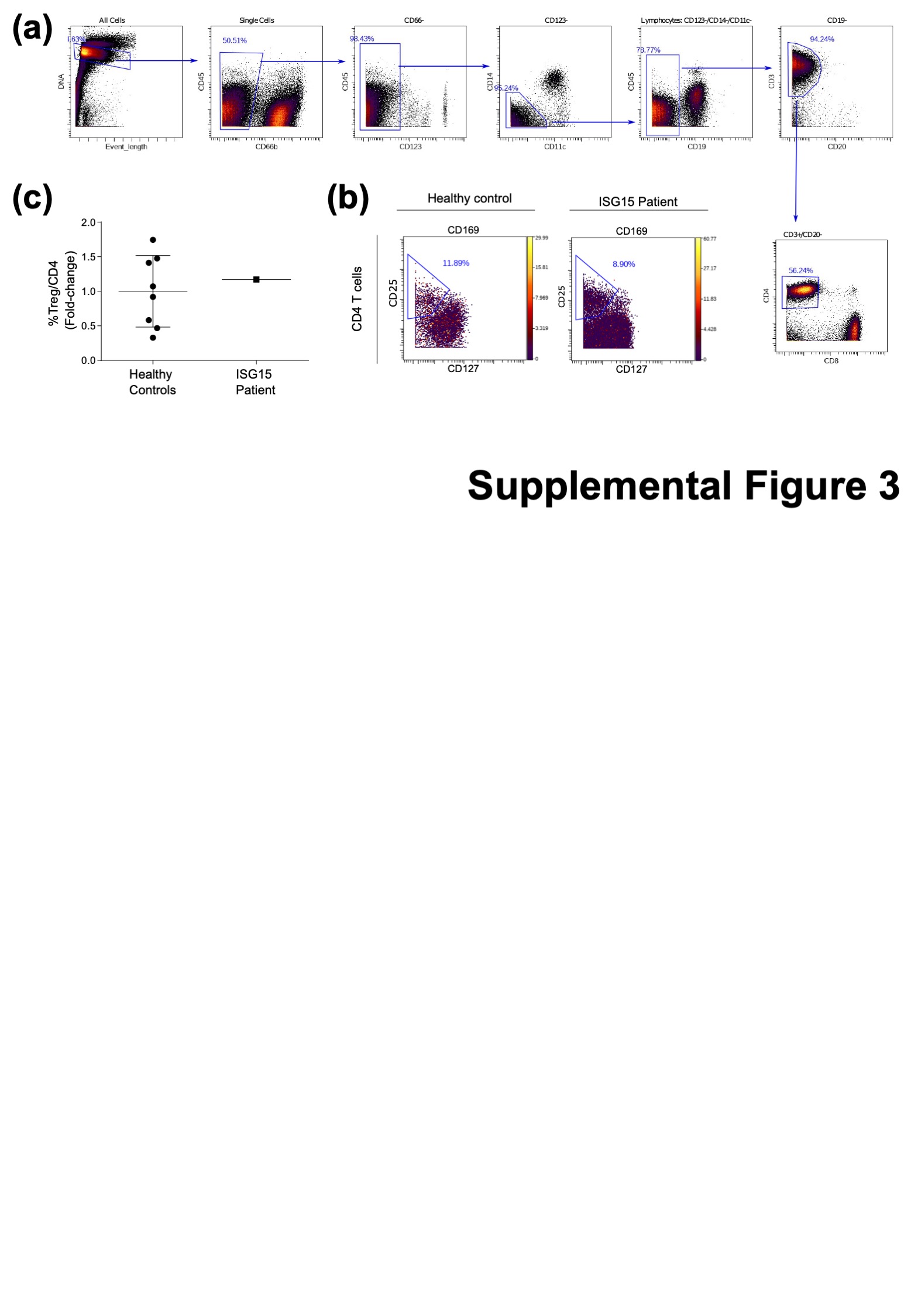


**Supplementary figure 3. CyTOF analysis of PBMCs does not reveal differences between ISG15-/- and healthy controls Treg counts.**

**(a**) Representative gating strategy of Tregs. **(b, c)** Tregs from manually gated CyTOF populations of 8 separate healthy controls and the ISG15^-/-^ patient’s PBMCs were quantified as the percentage of CD4^+^ T cells and expressed as fold-change (patient/average controls).


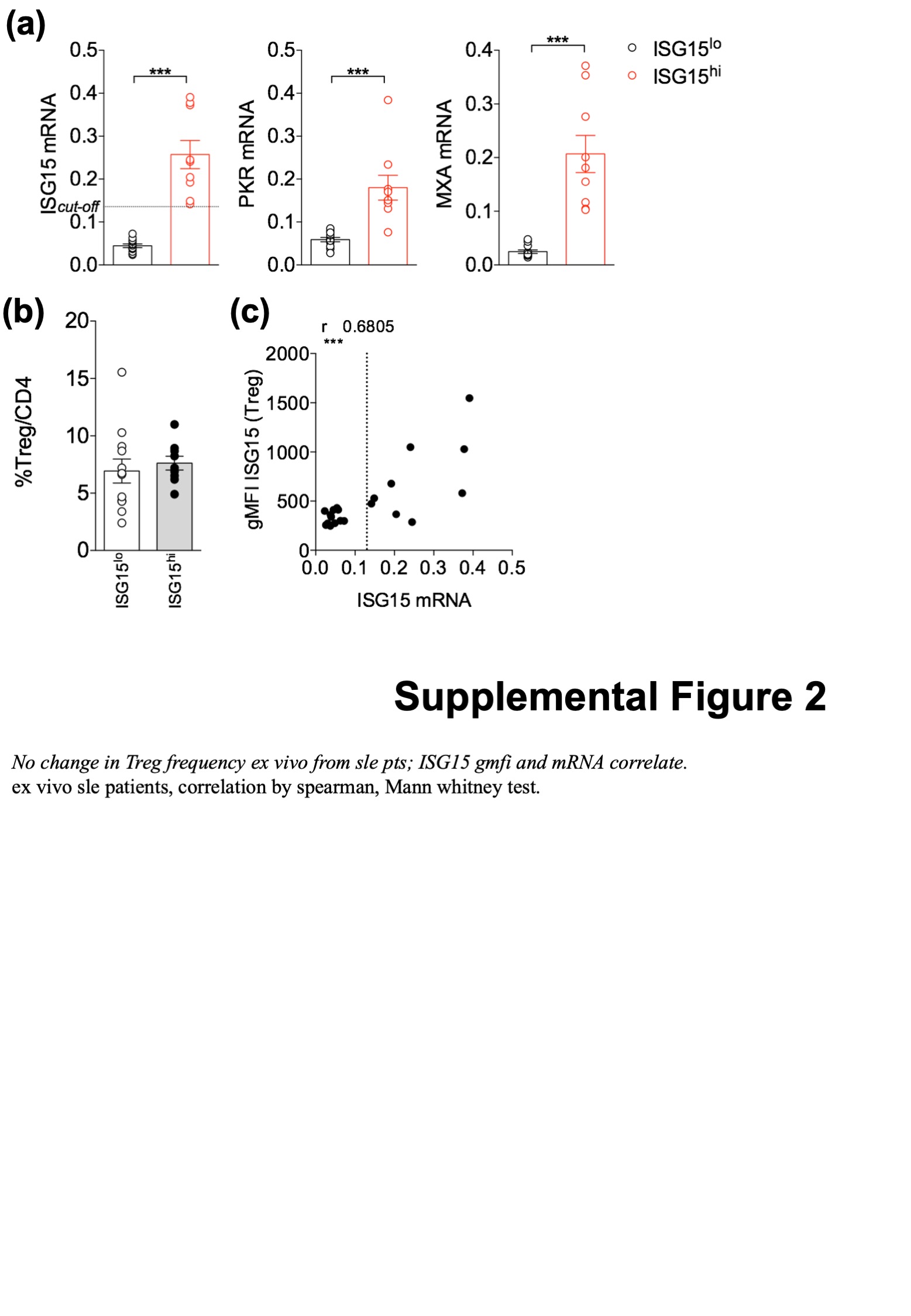


**Supplementary figure 4. ISG15 mRNA levels in PBMCs of SLE patients do not correlate with Treg frequency measured *ex vivo.***

**(a)** Real-time PCR for *ISG15*, *PKR* and *MXA* was performed in PBMCs from 21 SLE patients, and patients were stratified into ISG15^lo^ (n = 12) and ISG15^hi^ (n = 9) using the average ISG15 expression level (0.135) as cutoff. Means ± SEM of *ISG15*, *PKR* and *MXA* expression levels (2^-ΔCt relative to *GAPDH*) are shown in the two subgroups. *** *P* < 0.005, by the Mann-Whitney *U-*test. **(b)** Treg frequency was estimated as the percentage of FOXP3^+^ CD127^lo^ T cells in gated live CD4 T cells from the PBMCs of SLE patients, stratified into ISG15^lo^ and ISG15^hi^ as indicated above. **(c)** Spearman correlation between *ISG15* mRNA expression in total PBMCs and ISG15 protein content (assessed as gMFI) in gated Tregs from SLE patients. The dotted line indicates the cut-off between ISG15^lo^ and ISG15^hi^ patients. *** *P* < 0.005.


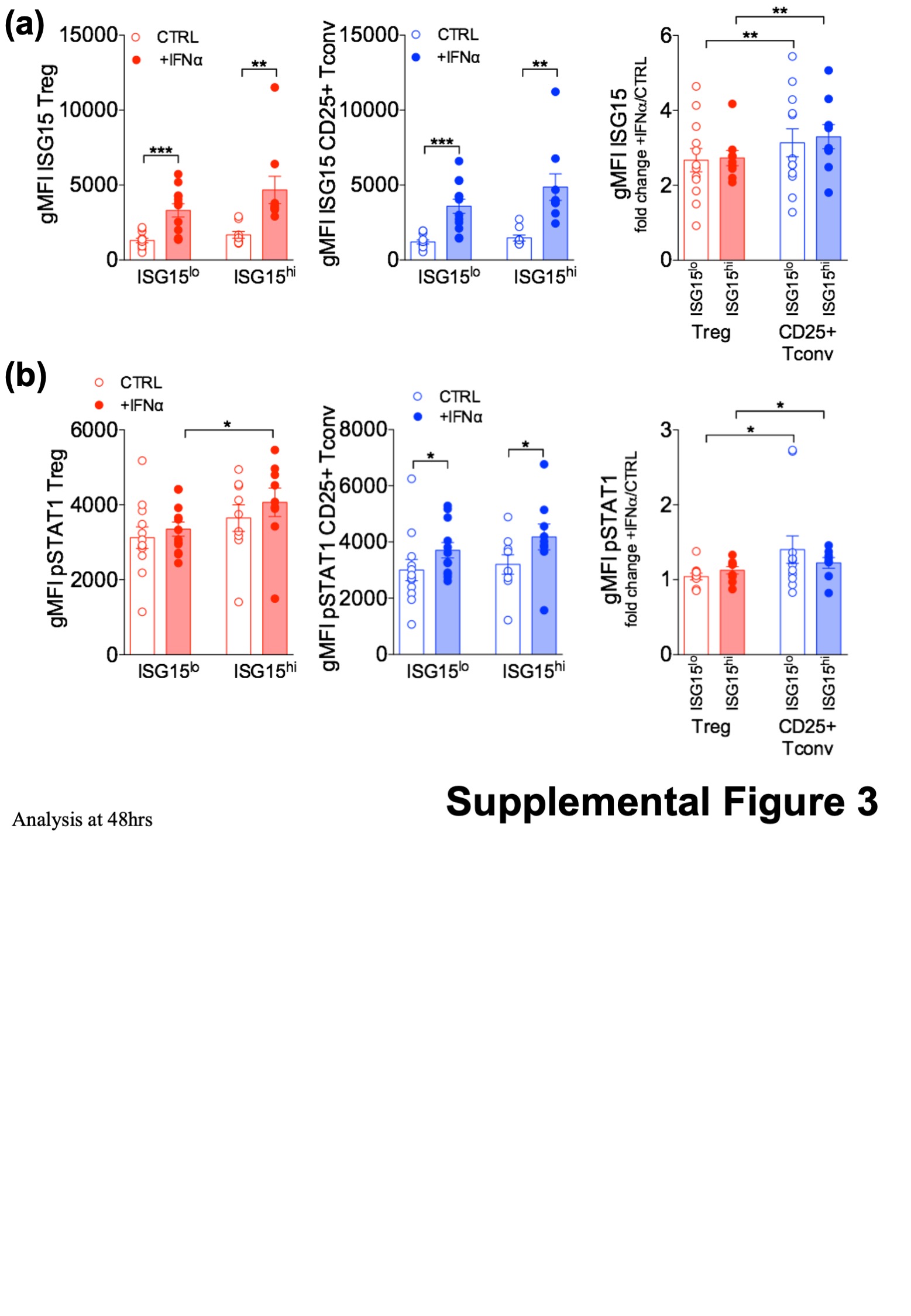


**Supplementary figure 5. Similar ISG15 and pSTAT1 levels in cells from ISG15^lo^ and ISG15^hi^ SLE patients after *in vitro* culture.**

SLE patients were stratified into ISG15^lo^ and ISG15^hi^, and PBMCs were stimulated for 48 hours with anti-CD3 alone (CTRL) or plus recombinant human IFNα (10^4 IU mL^-1^); then, intracellular content of ISG15 **(a)** or pSTAT1 **(b)** was assessed in gated Tregs and CD25^+^ Tconvs by flow cytometry. Means ± SEM of gMFI values, or their fold changes (as the +IFNα/CTRL ratio), are shown in the indicated patient subgroups and conditions. * *P* < 0.05, ** *P* < 0.01, *** *P* < 0.005, by the Mann-Whitney *U-*test between ISG15^lo^ and ISG15^hi^ patients, and by the Wilcoxon matched-pairs test between Tregs and Tconvs or between CTRL and +IFNα.


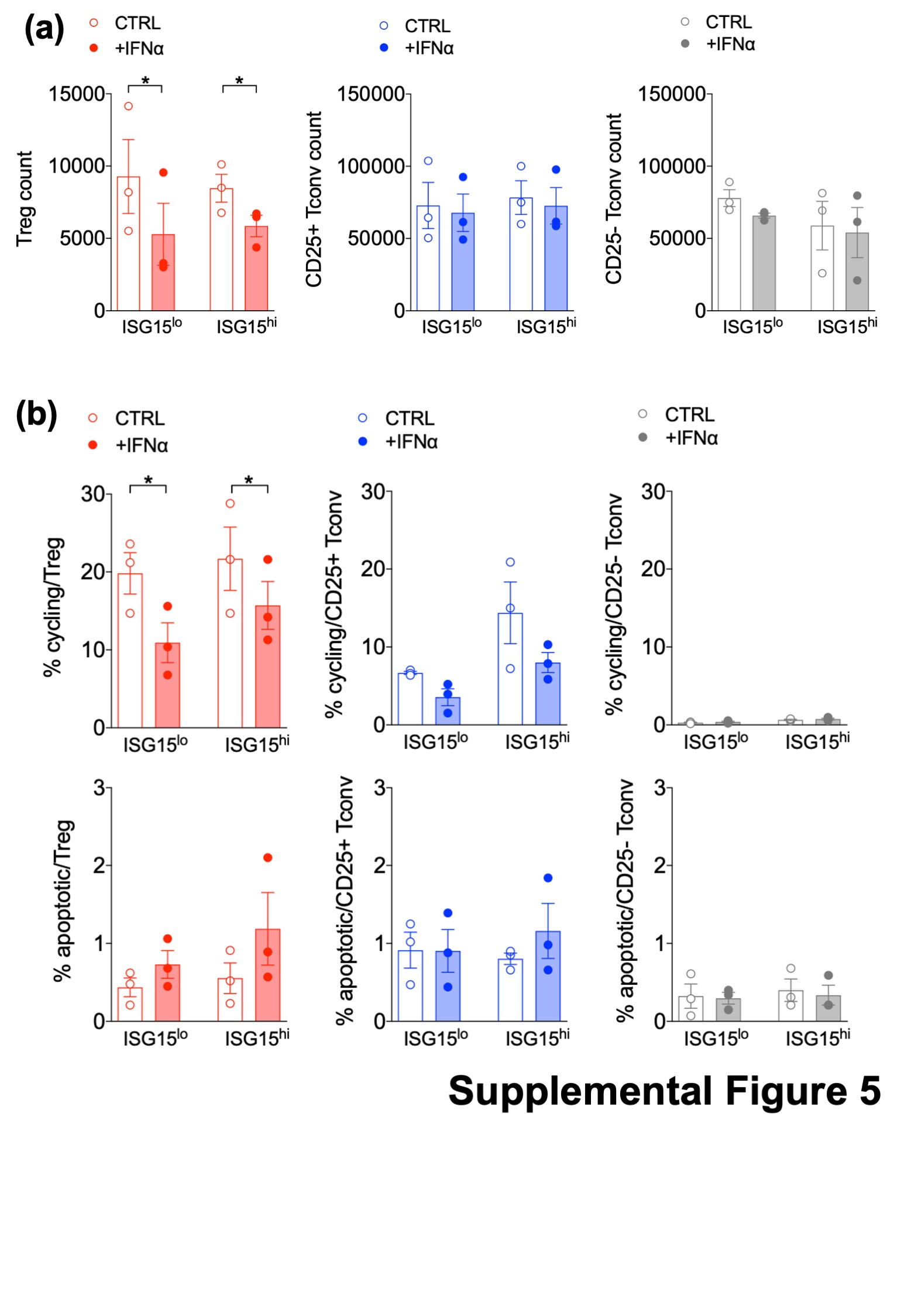


**Supplementary figure 6. IFNα exerts an antiproliferative effects on Tregs from SLE patients after *in vitro* culture.**

**(a)** PBMCs isolated from SLE patients (n = 6), of which 3 ISG15^hi^ and 3 ISG15^lo^, were cultured 48 hours with anti-CD3 alone (CTRL) or plus recombinant human IFNα (10^4 IU mL^-1^), then the recovered cells were manually counted. The frequencies of the indicated subsets in gated live single lymphocytes were analyzed by flow cytometry and their absolute counts were then calculated. **(b)** Means ± SEM of the percentages of Ki67+ (cycling) and AnnexinV+ (apoptotic) cells in gated Tregs, activated (CD25+) Tconvs and resting (CD25^-^) Tconvs are shown. * *P* < 0.05, by the paired *t*-test.
